# Modelling of Grain Growth Kinetics in Porous Ceramic Materials under Normal and Irradiation Conditions

**DOI:** 10.3390/ma2031252

**Published:** 2009-09-10

**Authors:** Mikhail S. Veshchunov

**Affiliations:** Nuclear Safety Institute (IBRAE), Russian Academy of Sciences B. Tul'skaya 52, 115191, Moscow, Russia; E-Mail: vms@ibrae.ac.ru; Tel. +7(495) 955-22-18; Fax: +7(495) 958-00-40

**Keywords:** porous ceramics, grain growth, intergranular bubble and pores, irradiated UO_2_ fuel

## Abstract

Effect of porosity on grain growth is both the most frequent and technologically important situation encountered in ceramic materials. Generally this effect occurs during sintering, however, for nuclear fuels it also becomes very important under reactor irradiation conditions. In these cases pores and gas bubbles attached to the grain boundaries migrate along with the boundaries, in some circumstances giving a boundary migration controlled by the movement, coalescence and/or sintering of these particles. New mechanisms of intergranular bubble and pore migration which control the mobility of the grain boundary under normal and irradiation conditions are reviewed in this paper.

## 1. Introduction

The interest in controlling grain growth in ceramic fabrication processes generally arises from two main causes. It may be a direct end in itself, insofar as the grain size of the finished article is one of the major factors determining its properties; alternatively it may be a means to the end of preparing articles of close to theoretical density. As a consequence, the justification for efforts to control grain size is sought in the advantage brought either by a specific grain size or by high density.

Grain size can affect the properties of the finished article. While few properties of ceramics are completely independent of grain size, most attention has been drawn to those mechanical and dielectric/magnetic properties where the structure-property relationship is very clear. The most striking of these are the effect of grain size $d_{gr}$ on fracture strength, $\sigma =f\left(d_{gr}^{-1/2}\right)$ [1,2], the effect of grain size on high temperature creep deformation, $\dot{\varepsilon }=f\left(d_{gr}^{-m}\right)$, where *m* is a constant whose value depends on the creep mechanism [3]. A wide range of electrical and magnetic parameters are affected by grain size [4,5].

The second reason for controlling grain growth has been found in the search for high density in sintering [6]. The recognition that densification can only proceed at a reasonable rate as long as the sources and sinks for the associated diffusion process are kept close together and, more particularly, the identification of the grain boundary and the pore as the sources and sinks for the diffusion atoms, have suggested that ultimate density is only to be expected where pores remain attached to grain boundaries [7].

Similarly to many other ceramic materials, nuclear fuel based on UO_2_ and (U,Pu)O_2_ is processed by powder sintering. However, in this case the main reason for controlling grain growth is to reduce the fission gas release in the fuel rods, in order to achieve increases in fuel burnup. With this in mind, the processing of ‘coarse grain’ microstructures was considered as early as the 1970’s with the aim of reducing the fraction of gas released by increasing the diffusion distances to the grain boundaries. Positive results were reported for coarse-grained UO_2_, obtained by annealing [8]. Grain growth in the final stage of sintering is the result of interactions between the grain boundaries and the residual porosity.

In addition to the atomic diffusion, a rather important mechanism of fission gas release from the interior of fuel grains to the grain boundaries under irradiation conditions is collection of gas lodged in the fuel matrix by moving grain boundaries. Incorporation of fission gas in a grain boundary by those mechanisms is irreversible because the thermodynamic solubility of the rare gases in UO_2_ is essentially small. Fission products in the grain boundaries migrate along the boundaries and precipitate into gas bubbles which eventually link up and vent to the environment. The latter phenomenon is important at high burnup, but the former process is dominant at low burnup resulting in significant growth of the bubbles and fuel swelling (reducing the thermal conductivity of the fuel material). 

Grain growth is the process by which the mean grain size of aggregates of crystals increases. The driving force for this process results from the decrease in free energy which accompanies reduction in total grain boundary area. Second-phase inclusions act as pinning agents to grain boundaries since the attachment of an inclusion reduces the total boundary energy by an amount equal to the specific surface energy times the area occupied by the inclusions. If the inclusions are relatively immobile, a boundary pinned at an inclusion can only move by breaking free. This occurs when the driving force for the boundary migration exceeds the pinning force exerted by inclusions on the boundary. In the case of mobile second-phase inclusions (e.g. gas bubbles or sintering pores), they migrate along with the boundaries, in some circumstances giving a boundary migration rate controlled by the movement of the second-phase particles. 

Burke and Turnbull [9] deduced a parabolic relationship for normal grain growth kinetics. They modelled migration of a boundary as occurring by atom transport across the boundary due to a surface curvature and pressure gradient between grains. In this approach the driving force applied to the boundary of a spherical grain with radius $R_{gr}$ is written as:

(1) $$\Delta G=\frac{\xi \gamma_{gb}}{R_{gr}}$$

where $\gamma_{gb}$ is the surface energy of the boundary, and ξ ≈ 1−2 is a geometric factor. Under simplifying assumption $R_{gr}={\bar{R}}_{gr}$, where ${\bar{R}}_{gr}$ is the mean grain radius, the mean grain boundary velocity is given by equation:

(2) $$v_{gb}^{\left(0\right)}=\frac{d{\bar{R}}_{gr}}{dt}=u_{gb}\Delta G=\frac{M^{\prime }}{{\bar{R}}_{gr}}$$

where $u_{gb}$ is the grain boundary mobility, and $M^{\prime }=u_{gb}\gamma_{gb}\xi$. After integration, Equation (2) results in the parabolic grain growth.

A more appropriate treatment of the grain growth problem with consideration of grain coalescence was performed by Greenwood [10], who modified Equation (1) to the form:

(3) $$\Delta G=\xi \gamma_{gb}\left(\frac{1}{R_{c}}-\frac{1}{R_{gr}}\right)$$

where $R_{c}$ is the critical radius which varies with time. Therefore the grain boundary velocity of a spherical grain with the radius $R_{gr}$ is given by equation:

(4) $$v_{gb}^{\left(0\right)}=\frac{dR_{gr}}{dt}=u_{gb}\Delta G=M^{\prime }\left(\frac{1}{R_{c}}-\frac{1}{R_{gr}}\right)$$

Grains grow or collapse depending on whether $R_{gr}>R_{c}$ or $R_{gr}<R_{c}$, respectively. 

Using Equation (4), the kinetics become identical with those for Ostwald ripening of a distribution of second phase particles, with interphase reactions controlling the rate at which large particles grow at the expense of smaller ones. Hillert [11] used previous analysis of Lifshitz and Slyozov [12] for Ostwald ripening to obtain parabolic kinetics for grain growth. According to Hillert's theory [11] the critical radius satisfies equation:

(5) $$\frac{dR_{c}}{dt}=\frac{M^{\prime }}{8}\frac{1}{R_{c}}$$

whereas the mean grain radius ${\bar{R}}_{gr}$ is related to the critical radius $R_{c}$ by:

(6) $${\bar{R}}_{gr}=\left(8/9\right)R_{c}$$

So, for the mean grain growth velocity this results in:

(7) $${\bar{v}}_{gb}^{\left(0\right)}=\frac{d{\bar{R}}_{gr}}{dt}=M\frac{1}{{\bar{R}}_{gr}}$$

where

(8) $$M=\frac{8}{81}M^{\prime }=\frac{8}{81}u_{gb}\gamma_{gb}\xi$$

Hence, in comparison with the simplified approach [10], the effective mobility of the mean grain boundary migration in Equation (7) turns out to be one order of magnitude smaller than in Equation (2). 

Speight and Greenwood [13] applied the grain growth theory to nuclear fuels taking into consideration the sweeping of entrapped gas by the front of an advancing grain boundary. The basic postulate of their model is that small bubbles, because they exert a minimal drag force on an advancing grain surface, are swept along with the moving boundary, whereas large bubbles, because of their higher drag, can detach from the advancing surface. 

In order to calculate the retarding effect of bubbles or pores on a separately moving grain boundary, Nichols [14] showed that in presence of the attached bubbles (or pores) the grain boundary motion is governed by the net force $\Delta G-Fn_{b}$, where *F* is the force applied to a separate bubble and $n_{b}$ is the surface concentration of bubbles, moving along with the grain boundary. Therefore the grain boundary velocity was calculated modifying Equation (2) as:

(9) $$v_{gb}=u\left(\Delta G-Fn_{b}\right)=\frac{v_{gb}^{\left(0\right)}}{\Delta G}\left(\Delta G-Fn_{b}\right)$$

Simultaneously, the bubble velocity $v_{b}$ is equal to $v_{gb}$, until the bubble is attached to the boundary [9]:

(10) $$v_{b}=bF=v_{gb}$$

where $b=2D_{b}/kT$, and $D_{b}$ is the bubble diffusion coefficient (dependent on the bubble radius $R_{b}$).

Using Equations (9) and (10) the force *F* can be calculated as:

(11) $$F=\frac{v_{gb}^{\left(0\right)}\Delta G/n_{b}}{v_{gb}^{\left(0\right)}+b\Delta G/n_{b}}$$

Therefore, one derives the equation for the grain boundary velocity [14]:

(12) $$v_{gb}=\frac{v_{gb}^{\left(0\right)}b\Delta G/n_{b}}{v_{gb}^{\left(0\right)}+b\Delta G/n_{b}}=\frac{u_{gb}b/n_{b}}{u_{gb}+b/n_{b}}\Delta G$$

or

(13) $$v_{gb}=v_{gb}^{\left(0\right)}{\left(1+\frac{v_{gb}^{\left(0\right)}}{\Delta G}\cdot \frac{n_{b}}{b}\right)}^{-1}$$

where $v_{gb}^{\left(0\right)}=u_{gb}\Delta G$ is the grain boundary velocity in the lack of the attached bubbles. 

However, Nichols analysed a simplified problem of a single boundary movement representing an average behaviour of an aggregate of crystals, without consideration of a real size distribution of grains and their coalescence. Such a consideration can be done in the framework of Hillert’s mean-field approach [11] and was performed in the author paper [15]:

(14) $${\bar{v}}_{gb}=\frac{d{\bar{R}}_{gr}}{dt}=v_{gb}^{\left(0\right)}{\left(1+\frac{81v_{gb}^{\left(0\right)}{\bar{R}}_{gr}}{8\xi \gamma_{gb}}\cdot \frac{n_{b}}{b}\right)}^{-1}$$

It is important to note from Equation (14) that pore (bubble) parameters control boundary movement when $81v_{gb}^{\left(0\right)}{\bar{R}}_{gr}/8\xi \gamma_{gb}>>b/n_{b}$. Comparing Equation (14) with Equation (13) one can see that in the advanced model (with application of Hillert’s approach to consideration of grain size distribution) this occurs significantly earlier when ${\bar{R}}_{gr}>>0.1\left(b/n_{b}\right)\xi \gamma_{gb}/v_{gb}^{\left(0\right)}$, *i.e.*, at grain size one order of magnitude smaller than in the simplified approach [14]. 

To take into account different kinds of gas bubbles on the grain boundary, *i.e.*, face (*f*), edge (*e*) and corner (*c*) bubbles, relationship similar to Equation (10) should be applied to each kind of porosity [16]:

(15) $$v_{gb}=v_{f}=b_{f}F_{f}=v_{e}=b_{e}F_{e}=v_{c}=b_{c}F_{c}$$

therefore, the net force acting on the boundary takes the form:

(16) $$\Delta G-n_{f}F_{f}-n_{e}F_{e}-n_{c}F_{c}=\Delta G-F_{f}\left(n_{f}+n_{e}\frac{b_{f}}{b_{e}}+n_{c}\frac{b_{f}}{b_{c}}\right)$$

and for the grain boundary velocity one obtains [15]:

(17) $${\bar{v}}_{gb}=\frac{d{\bar{R}}_{gr}}{dt}=v_{gb}^{\left(0\right)}{\left(1+\frac{81v_{gb}^{\left(0\right)}{\bar{R}}_{gr}}{8\xi \gamma_{gb}}\cdot \left(n_{f}b_{f}^{-1}+n_{e}b_{e}^{-1}+n_{c}b_{c}^{-1}\right)\right)}^{-1}$$

In the absence of porosity the grain growth under isothermal conditions can be satisfactorily described by parabolic kinetics, derived by direct integration of Equation (7):

(18) $${\bar{R}}_{gr}^{2}(t)-{\bar{R}}_{gr}^{2}(0)=K\left(T\right)\;t$$

where ${\bar{R}}_{gr}(0)$ and ${\bar{R}}_{gr}(t)$ are the average grain radii of the sample before annealing and after an annealing time *t* at a temperature *T*, respectively. 

In porous materials the grain growth is the result of interactions between grain boundaries and pores, which give rise to drag effect which impedes boundary motion in accordance with Equation (14). For instance, for materials with the total porosity invariable during annealing the grain growth was approximated in [14,17] by a more slow kinetic equation:

(19) $${\bar{R}}_{gr}^{n}(t)-{\bar{R}}_{gr}^{n}(0)=K\prime t$$

with the growth exponent *n* = 3 or 4, depending on pore migration mechanism, in a good agreement with experimental observations, e.g. [18,19]. However, recently it was revealed that in many cases the normal grain growth kinetics must be described by non-integer exponents, somewhat different from 3 or 4 [20].

In Section 2 it is shown, following the original publication of the author [21], that additional consideration of the porous material densification (*i.e.*, porosity reduction under high temperature annealing conditions) in the course of the grain growth allows explanation of complicated grain growth kinetics characterised by non-integer growth exponents observed in the tests.

In application to irradiated materials with gas bubbles formed on the grain boundaries, Nichols’ approach [14] has another deficiency associated with consideration of a retarding effect using the standard mechanisms of bubble mobility derived by Shewmon [22] for spherical (e.g. intragranular) bubbles. However, besides a more complicated (so called “lenticular”) shape of grain face bubbles, the migration mechanism of these bubbles might be essentially different from that of the intragranular bubbles, owing to their specific location on and interaction with a grain boundary. A new mechanism of the lenticular grain face bubble migration which controls the bubble mobility and determines the drag force exerted on the grain boundary, proposed in the author’s paper [15], will be presented in Section 3. 

In Section 4 of the current paper the new mechanism is extended to consideration of the grain boundary peripheral (edge and corner) bubbles migration associated with vacancy fluxes along the grain boundary, following the original publication of the author [23].

In Section 5 further generalization and improvement of the model for the grain growth controlled simultaneously by sintering pores and gas bubbles migration (also considered in [23]), is presented. For this purpose, pore coalescence during grain growth and pores shrinkage, caused by vacancies thermal evaporation from pores and by vacancies knockout from pores under irradiation, are self-consistently considered in the improved model. 

Implementation of the advanced grain growth model in the MFPR code designed for modelling fuel performance and fission products release [24,25], and its validation against various tests are presented in Section 6.

## 2. Effect of Sintering Pores on Normal Grain Growth Kinetics

**Figure 1:**
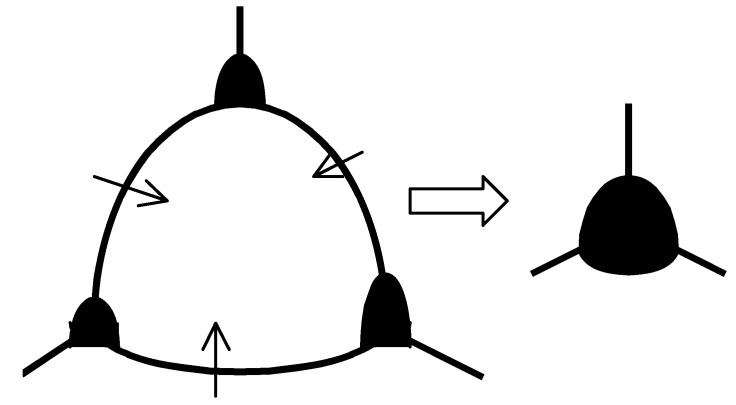
Pore migration with boundaries and resulting pore growth during normal grain growth.

For explanation of the normal grain growth kinetics, Equation (19), a series of models has been proposed, most of them based on consideration of Kingery and François [26]. They assumed that, as grains are removed in the growth process, pores migrating with the boundaries are brought together, and pore growth occurs together with grain growth, see Figure 1. After, say, twofold increase of the mean grain size, an amount of grains $N_{gr}$ decreases by one order of magnitude ($N_{gr}\propto {\bar{R}}_{gr}^{-3}$), and practically all pores are located at grain corners, so amount of pores $N_{p}$ becomes proportional to the amount of grains, $N_{p}\propto N_{gr}$.

Kingery and François additionally assumed that in the later stages of sintering grain growth is relatively faster than fuel densification, so that the pore fraction remains essentially constant. Under such an assumption, the mean pore radius ${\bar{R}}_{p}\propto N_{p}^{-1/3}$ and thus is proportional to the mean grain radius:

(20) $${\bar{R}}_{p}\propto {\bar{R}}_{gr}$$

On this basis, to account for drag effect of the pores, they proposed to introduce an additional factor ${\bar{R}}_{p}^{-1}\propto {\bar{R}}_{gr}^{-1}$ in the r.h.s of Equation (7), leading to Equation (19) with *n* = 3 after integration.

Later Nichols [14] derived a more appropriate equation for the drag force, Equation (12), which was applied by Brook [17] to consideration of normal grain growth kinetics controlled by pore migration. Using this equation for the later stages of sintering when corner pore mobility controls boundary movement, Brook obtained an equation:

(21) $$\frac{d{\bar{R}}_{gr}}{dt}\propto \frac{b_{p}}{{\bar{R}}_{gr}n_{p}}$$

where $n_{p}$ is the surface concentration of pores on the boundary, and pore mobility:

(22) $$b_{p}\propto {\bar{R}}_{p}^{-n}$$

is determined by migration mechanism, *i.e.*, *n* = 3 for the mechanisms of lattice diffusion and gas phase transport at *P = const*., and *n* = 4 for the surface diffusion mechanism [22]. He also noticed that for the considered situation where pores are located at grain corners, their separation is proportional to ${\bar{R}}_{gr}$, so the surface concentration of pores on the boundary is inversely proportional to ${\bar{R}}_{gr}^{-2}$:

(23) $$n_{p}\propto {\bar{R}}_{gr}^{-2}$$

Substitution of Equations (22) and (23) in Equation (21) results in:

(24) $$\frac{d{\bar{R}}_{gr}}{dt}\propto {\bar{R}}_{gr}^{1-n}$$

or, after its integration:

(25) $${\bar{R}}_{gr}^{n}(t)-{\bar{R}}_{gr}^{n}(0)=K\prime t$$

Therefore, namely the pore migration mechanisms by lattice diffusion and gas phase transport at *P = const*. provide grain growth kinetics with *n* = 3, whereas *n* = 4 is afforded by the surface diffusion mechanism. 

Nevertheless, Bourgeois *at al*. [20] noticed that to describe grain growth during porous UO_2_ thermal treatment (sintering) in their recent tests, the slope $\ln\left({\bar{R}}_{gr}^{n}(t)-{\bar{R}}_{gr}^{n}(0)\right)$ as a function of lnt might be quite different from 1, whether with *n* = 3 or *n* = 4, and also from one temperature to another. Therefore, in order to describe grain growth in these cases, a non-integer exponent *n* must be used. 

In order to explain such behaviour, one should take into account that Equation (25) was derived under simplifying assumption that the pore fraction remains essentially constant during grain growth, $N_{p}{\bar{R}}_{p}^{3}=const.$ However, this assumption was not confirmed in the new tests [20]. Indeed, in these tests changes in density of fuel pellets during heat treatment were monitored simultaneously with the grain growth measurements, which demonstrated a plain correlation between grain growth and fuel densification.

In order to take into consideration shrinkage of isolated pores owing to vacancies evaporation during thermal annealing, Speight and Beere’s approach [27] (for the grain face cavities) was applied in [21] to the case of the corner pore located at intersection of 6 grain faces (4 hexagonal and 2 square) of UO_2_ grains considered as truncated octahedron (see details of this structure in Section 4):

(26) $$\frac{\partial V_{p}}{\partial t}\approx -\frac{3.67\pi \Omega D_{gb}w}{\beta kT}\frac{2\gamma_{s}}{R_{p}}$$

Where *D_gb_* is the grain boundary self-diffusion coefficient of uranium atoms, 2*w* is the thickness of the grain boundary, $\gamma_{s}$ is the surface tension of the pore, $\beta =\ln(R_{c}/R_{p})-0.25(1-R_{c}^{2}/R_{p}^{2})(3-R_{c}^{2}/R_{p}^{2})$ is the dimensionless factor, ${\left(\pi R_{c}^{2}\right)}^{-1}=n_{p}$ determines the radius of the sink free zone $R_{c}$, which can be estimated taking into account that 24 corners are distributed over the grain surface with the area of ≈ $4\pi R_{gr}^{2}$, *i.e.*,

(27) $$n_{p}\approx 6/\pi R_{gr}^{2}\;\mathrm{and}\;R_{c}\approx R_{gr}/\sqrt{6}$$

Therefore, decrease of an isolated pore radius can be evaluated from Equations (26)-(27) as:

(28) $$\frac{\partial R_{p}}{\partial t}\approx -\frac{5.5\pi \Omega D_{gb}w}{\beta kT}\frac{\gamma_{s}}{R_{p}}\equiv -\frac{\alpha }{R_{p}}$$

where

(29) $$\alpha =\frac{5.5\Omega D_{gb}w\gamma_{s}}{\beta kT}$$

Neglecting pores shrinkage, one can obtain (following Kingery and François [26]) that variation of total porosity in the course of pores coalescence is zero, $d\left(N_{p}{\bar{V}}_{p}\right)/dt=0$. However, taking pores shrinkage into consideration, one will obtain that in this case

(30) $$\frac{d\left(N_{p}{\bar{V}}_{p}\right)}{dt}=N_{p}\frac{\partial {\bar{V}}_{p}}{\partial t}$$

where

(31) $$\frac{d\left(N_{p}{\bar{V}}_{p}\right)}{dt}=N_{p}\frac{d{\bar{V}}_{p}}{dt}+{\bar{V}}_{p}\frac{dN_{p}}{dt}$$

and

(32) $$N_{p}\propto N_{gr}\propto {\bar{R}}_{gr}^{-3}$$

Here $\partial {\bar{V}}_{p}/\partial t$ denotes variation of the pore mean volume owing solely to pores shrinkage, Equation (26), whereas $d{\bar{V}}_{p}/dt$ denotes total variation of the pore mean volume owing to pores simultaneous shrinkage and coalescence [21].

Substituting Equations (26), (31) and (32) in Equation (30), one obtains

(33) $$\frac{d{\bar{R}}_{p}}{dt}-\frac{{\bar{R}}_{p}}{{\bar{R}}_{gr}}\frac{d{\bar{R}}_{gr}}{dt}=-\frac{\alpha }{3{\bar{R}}_{p}^{3}}$$

In the case when the corner pore mobility controls grain boundary movement, the relationship for the mean grain radius growth controlled by pore mobility, similar to Equation (14), takes the form:

(34) $$\frac{d{\bar{R}}_{gr}}{dt}\approx \frac{8}{81}\frac{\gamma_{gb}\xi }{{\bar{R}}_{gr}}\frac{b_{p}}{n_{p}}\approx 0.1\frac{\gamma_{gb}\xi }{{\bar{R}}_{gr}}\frac{b_{p}\pi {\bar{R}}_{gr}^{2}}{6}$$

where Equation (27) was used for $n_{p}$, and $b_{p}\approx \frac{3D_{s}\Omega^{4/3}}{2\pi kTR_{p}^{4}}$ [22], if the surface diffusion mechanism controls the pore migration kinetics. Substituting this value in Equation (34), finally one obtains:

(35) $$\frac{d{\bar{R}}_{gr}}{dt}=\varphi \frac{{\bar{R}}_{gr}}{{\bar{R}}_{p}^{4}}$$

where $\varphi \approx \frac{\gamma_{gb}\xi }{40}\frac{D_{s}w\Omega }{kT}$.

The system of Equations (33) and (35) has the solution:

(36) $${\bar{R}}_{p}\propto {\bar{R}}_{gr}^{\left(1-\alpha /3\varphi \right)}$$

and

(37) $${\bar{R}}_{gr}^{4\left(1-\alpha /3\varphi \right)}\left(t\right)-{\bar{R}}_{gr}^{4\left(1-\alpha /3\varphi \right)}\left(0\right)=Kt$$

The total porosity reduction can be calculated as $V_{pores}=N_{p}{\bar{V}}_{p}\propto N_{p}{\bar{R}}_{p}^{3}\propto {\left({\bar{R}}_{p}/{\bar{R}}_{gr}\right)}^{3}$ and after substitution of Equation (36):

(38) $$V_{pores}=N_{p}{\bar{V}}_{p}\propto {\bar{R}}_{gr}^{-\alpha /\varphi }={\bar{R}}_{gr}^{-a}$$

where a=α/φ.

Substituting Equations (29) and (37) in Equation (38), one can evaluate:

(39) $$a=\alpha /\varphi \approx \frac{220}{\xi \beta }\frac{D_{gb}}{D_{s}}$$

where ξ≈ 1−2, parameter *β* depends on the fuel porosity (see designations after Equation (26)) and for the fuel density 96-98% varies in the range 0.2–0.3. Numerical estimations of Equation (39) presented in [21] are in a reasonable agreement with the exponents derived from the measured in [20] correlations between grain growth and fuel densification, a≈0.2−0.3. 

This means, as seen from Equation (37), that for the surface diffusion mechanism of pores the power exponent attains non-integer value ≈ 3.6–3.7, in a qualitative agreement with observations [20]. Direct comparison of calculation results with the measurements will be presented in Section 6.2.

## 3. Grain Growth Kinetics Controlled by Grain Face Bubble Migration

In accordance with [22], the mobility of a spherical intragranular bubble with radius $R_{b}$ is determined by various migration mechanisms:

(40) $$b\propto R_{b}^{-n}$$

where *n* = 3 for the mechanisms of lattice diffusion and gas phase transport, and *n* = 4 for the surface diffusion mechanism.

It was usually assumed that the same migration mechanisms can be also applied to the grain face bubbles with some renormalisation of the proportionality coefficient in Equation (40), owing to a more complicated lenticular form of these bubbles. However, a more profound difference from free intragranular bubbles arises on grain faces, which can significantly reduce the intergranular bubble mobility and thus migration velocity of the grain boundary. This new rate determining mechanism proposed in [15] of bubble migration will be presented in this Section.

### 3.1. Phenomenological Consideration

Before presenting a more detailed “microscopic” consideration of the grain boundary migration with attached gas-filled bubbles, a phenomenological approach to calculation of the retarding force exerted by bubbles on the moving boundary will be presented.

The driving force for the boundary migration can be derived from the pressure gradient across the boundary arising from its curvature given by expression $\Delta G=\xi \gamma_{gb}/R_{gr}$ (see Section 1). This pressure gradient between the two adjacent grains provides different boundary conditions also for gas bubbles in these grains; in particular, an additional external hydrostatic pressure $p_{ext}=\Delta G$ is applied to the spherical segment of the lenticular bubble surface in the shrinking grain.

**Figure 2:**
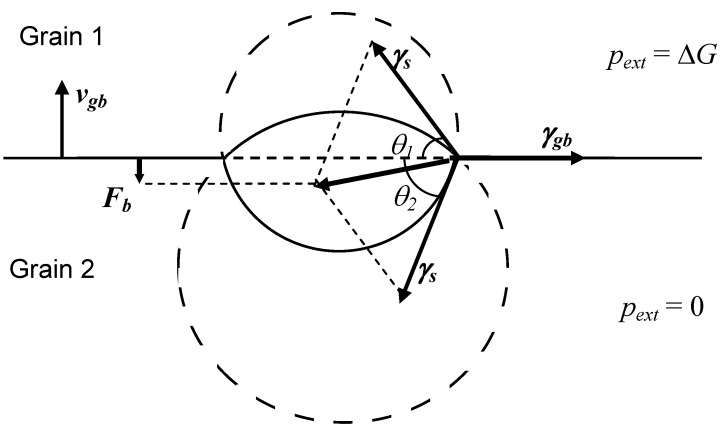
Determination of the drag force exerted by attached lenticular bubble on moving grain boundary.

In order to clarify the nature of the drag force exerted on the grain boundary by an attached bubble, at first a simplified limiting case of a complete equilibrium of the lenticular bubble with both grains (shrinking and growing) separated by the boundary under steady-state conditions, will be considered, Figure 2. In this limiting case:

(41) $$\Delta p_{2}\equiv p_{b}-2\gamma_{s}/R_{2}=0$$

(42) $$\Delta p_{1}\equiv p_{b}-2\gamma_{s}/R_{1}-\Delta G=0$$

where $p_{b}$ is the internal bubble pressure, $R_{1}$ and $R_{2}$ are the curvature radii of the two surface segments of the bubble.

One can see from Equations (41) and (42) that the curvature radii of the two bubble surfaces are different, this induces different contact angles $\theta_{1}$ and $\theta_{2}$ with the grain boundary:

(43) $$R_{1}=\sin\theta_{1}=R_{2}\cdot \sin\theta_{2}=\rho_{b},$$

where $\rho_{b}$ is the projected radius of the bubble in the plane of the boundary. 

Assuming a balance between the surface tension forces in the plane of the grain boundary under steady-state conditions:

(44) $$\gamma_{gb}=\gamma_{s}(\cos\theta_{1}+\cos\theta_{2})$$

one can calculate a net force exerting by the bubble on the grain boundary in the normal to the grain boundary direction (see Figure 2):

(45) $$2\pi \rho \gamma_{s}(\sin\theta_{2}-\sin\theta_{1})=F_{b}$$

Substituting Equations (41)–(44) in Equation (45), one gets:

(46) $$F_{b}=\Delta G\cdot \pi \rho_{b}^{2}$$

and therefore, in accordance with Equation (2), the driving force for the grain boundary migration is reduced proportionally to the reduction of the grain boundary area owing to its coverage with bubbles:

(47) $$\Delta G^{\prime }=\Delta G-F_{b}n_{b}=\Delta G(1-n_{b}\cdot \pi \rho_{b}^{2})$$

The above presented consideration of the bubble equilibrium with the two grains can be justified only in the case when the rate determining process of bubble mobility is infinitely fast in comparison with the grain boundary migration. In a more general case of a finite bubble mobility, a complete equilibrium between the bubble and the two grains is not attained, hence Equations (41) and (42) are not anymore valid. It is straightforward to show that in order to uphold a coherent migration of the grain boundary and the attached bubble in this case, the values $\Delta p_{1}$ and $\Delta p_{2}$ become non-zero and obey the relationship:

(48) $$\Delta p_{1}=p_{b}-\frac{2\gamma_{s}}{R_{1}}-\Delta G=-\Delta p_{2}=\frac{2\gamma_{s}}{R_{2}}-p_{b}=\varepsilon >0$$

**Figure 3:**
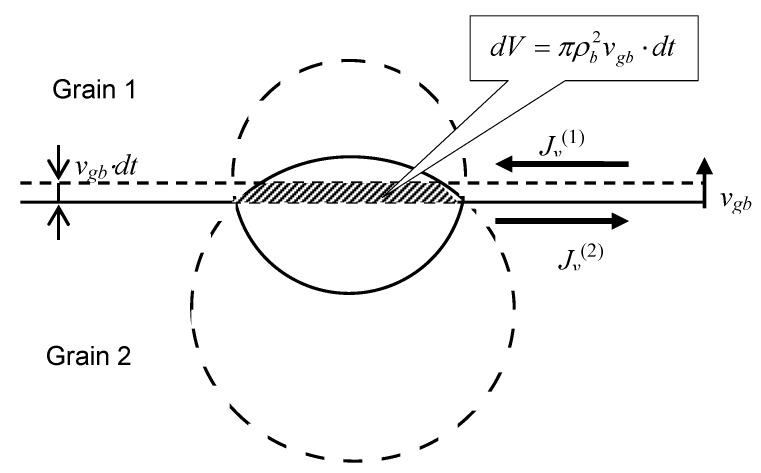
Determination of vacancy fluxes along the grain boundary in two adjacent grains providing relocation of a lenticular bubble coherently with the grain boundary.

Indeed, during a time interval dt the grain boundary moves over a distance $v_{gb}dt$. If the bubble is “frozen” at its position, the volume of the upper part of the bubble will be decreased by a value $dV=\pi \rho_{b}^{2}v_{gb}\cdot dt$, whereas the volume of the lower part will be increased by the same value dV, see Figure 3. 

In order to sustain the bubble migration with the grain boundary velocity $v_{gb}$, vacancy fluxes along the upper and lower surfaces of the grain boundary, $J_{v}^{\left(1\right)}$ and $J_{v}^{\left(2\right)}$, should compensate these volume variations:

(49) $$J_{v}^{\left(1\right)}2\pi \rho_{b}\Omega dt=-J_{v}^{\left(2\right)}2\pi \rho_{b}\Omega dt=dV=\pi \rho_{b}^{2}v_{gb}\cdot dt$$

where Ω is the vacancy volume. It is assumed that each of the vacancy fluxes ($J_{v}^{\left(1\right)}$ or $J_{v}^{\left(2\right)}$) occurs in a thin surface layer with a thickness *w* ≈ 0.5 nm of the corresponding grain (grain 1 or grain 2), characterised by a relatively high self-diffusion coefficient $D_{gb}$. 

These fluxes will be farther calculated in the following Section 3.2, nevertheless, from the physical point of view (confirmed by calculations presented below) it is clear that the values of $J_{v}^{\left(1\right)}$ and $J_{v}^{\left(2\right)}$ are determined by the pressure differences $\Delta p_{1}$ and $\Delta p_{2}$, respectively, which should obey condition $\Delta p_{1}=-\Delta p_{2}$, Equation (48), in order to sustain relationship $J_{v}^{\left(1\right)}=-J_{v}^{\left(2\right)}$, Equation (49). 

Equations (43)–(45) are still valid for the considered case of a non-equilibrium bubble with the steady-state lenticular shape, and along with Equation (48) determine the retarding force:

(50) $$F_{b}=2\pi \rho \gamma_{s}(\sin\theta_{2}-\sin\theta_{1})=\pi \rho_{b}^{2}(\Delta G+2\varepsilon )$$

Substitution Equation (50) in Equation (9) results in:

(51) $$v_{gb}=u_{gb}\lfloor \Delta G(1-n_{b}\cdot \pi \rho_{b}^{2})-2\varepsilon n_{b}\cdot \pi \rho_{b}^{2}\rfloor$$

Superposition of Equations (51) and Equation (49) with explicitly calculated fluxes $J_{v}^{\left(1\right)}$ and $J_{v}^{\left(2\right)}$ as a function of *ε* will finally determine the migration of the grain boundary with attached bubbles. 

The same result, Equation (51), derived in the present Subsection in phenomenological approach (*i.e.*, by consideration of mechanical forces, acting on the boundary and bubbles), can be obtained in a more accurate microscopic approach based on self-consistent calculation of vacancy fluxes across and along the grain boundary, which will be presented in the following Subsection 3.2. 

### 3.2. Microscopic Consideration

In accordance with Cole, Feltham and Gillam [28], migration of a grain boundary of a growing grain takes place in steps of one interatomic spacing *a* as atoms transfer from the neighbouring grain across the boundary under the pressure difference Δ*G* across the boundary:

(52) $$v_{gb}^{\left(0\right)}=\frac{2\upsilon a\Omega }{kT}\Delta G\exp(-\frac{Q}{kT})\equiv u_{gb}\Delta G$$

where *υ* is the atomic oscillation frequency on the grain boundary, *Q* is the activation energy for self-diffusion in the grain boundary, Ω is the atomic volume. The grain boundary mobility $u_{gb}=\frac{2\upsilon a\Omega }{kT}\exp(-\frac{Q}{kT})$, can be also evaluated following Burke and Turnbull [9] as:

(53) $$u_{gb}=\frac{D_{gb}\Omega }{2wkT}$$

where 2*w* ≈ 1 nm is the thickness of the grain boundary, $D_{gb}$ is the self-diffusion coefficient in the grain boundary.

The above described process of atomic jumps can be equivalently considered as translations of vacancies from the growing grain to the adjacent one with the same rate as translations of atoms in the opposite direction. The corresponding flux of vacancies ${\tilde{J}}_{v}^{\left(0\right)}$ in the normal to the grain boundary direction is uniform over the grain boundary surface (with the total area *S*) and thus determines the grain boundary relocation during the time interval dt, in accordance with the following relationship: ${\tilde{J}}_{v}^{\left(0\right)}\Omega S\cdot dt=S\cdot dx$. Therefore, the grain boundary migration velocity $v_{gb}^{\left(0\right)}=dx/dt$ can be represented in the form $v_{gb}^{\left(0\right)}={\tilde{J}}_{v}^{\left(0\right)}\Omega$, and thus:

(54) $${\tilde{J}}_{v}^{\left(0\right)}=u_{gb}\Delta G/\Omega$$

In the presence of attached bubbles with the surface coverage $n_{b}$ and mean projected radius $\rho_{b}$, the vacancy flux takes place across the reduced surface of the grain boundary $S\left(1-n_{b}\pi \rho_{b}^{2}\right)$. In the limiting case (corresponding to an infinite bubble mobility, or *ε* → 0), when the lenticular bubble attains equilibrium with both grains separated by the boundary, the vacancy flux is still uniform over the reduced grain boundary surface, and thus, Equation (54) can be used in the balance equation:

(55) $$v_{gb}^{\left(0\right)}S={\tilde{J}}_{v}^{\left(0\right)}\Omega S\left(1-n_{b}\pi \rho_{b}^{2}\right)$$

Therefore, in this case the grain boundary velocity is calculated as:

(56) $$v_{gb}^{\left(0\right)}=u_{gb}\Delta G\left(1-n_{b}\cdot \pi \rho_{b}^{2}\right)$$

in agreement with Equation (47).

In a more general case of a limited bubble mobility when a complete equilibrium between the bubble and the grains is not attained and *ε* > 0, a spatial variation of the vacancy chemical potential over the grain boundary faces takes place. On the one hand, this chemical potential variation induces the vacancy fluxes to (from) the bubble along the upper (lower) surface of the grain boundary, $J_{v}^{\left(1\right)}$ and $J_{v}^{\left(2\right)}$, introduced in Equation (49). On the other hand, the pressure drop across the boundary becomes also non-uniform over the grain face area. In order to calculate the total vacancy flux across the boundary in this case, one should self-consistently consider the vacancy transport along and across the grain boundary, on the base of calculation of the spatial variation of the vacancy chemical potential.

As shown by Speight and Beere [27], variation of the surface chemical potential $\mu (r)=\sigma_{nn}(r)\Omega$ in a grain reflects exactly the steady state distribution of normal stresses over the grain boundary area unoccupied by bubbles. In the currently considered problem with a moving grain boundary under pressure difference across the boundary, such a conclusion should be generalised and independently applied to each of the two adjacent grains, $\mu_{1,2}(r)=\sigma_{nn}^{\left(1,2\right)}(r)\Omega$. The integral of these stresses over the area (with the mean radius $R_{c}\approx {\left(\pi n_{b}\right)}^{-1/2}$) associated with one bubble must equal the total load applied to each face of the grain boundary. Hence, following [27], one obtains:

(57) $$\Omega^{-1}\int_{\rho_{b}}^{R_{c}}\mu_{1,2}(r)2\pi rdr=\sigma_{1,2}\pi R_{c}^{2}-(\frac{2\gamma_{s}}{R_{1,2}}-P_{b})\pi \rho_{b}^{2}$$

where the first term on the r.h.s. arises from the normal stresses $\sigma_{1,2}$ at each of two surfaces of the grain boundary in the absence of attached bubbles. In the presently considered case these stresses uphold the pressure gradient Δ*G* across the grain boundary, *i.e.*,

(58) $$\sigma_{2}=\sigma_{1}+\Delta G$$

The second term on the r.h.s. of Equation (57) expresses the force which the bubble surface tension exerts on the boundary. This term can be calculated as the integral of the normal stress on the lenticular bubble surface $\mu_{1,2}(R_{1,2},\theta )=\sigma_{nn}^{\left(1,2\right)}(\theta )\Omega =(\frac{2\gamma_{s}}{R_{1,2}}-P_{b})\Omega$ over the corresponding surface segment of the bubble: $\int \sigma_{nn}^{\left(1,2\right)}(R_{1,2})dS_{1,2}=(\frac{2\gamma_{s}}{R_{1,2}}-P_{b})\pi \rho_{b}^{2}$.

**Figure 4:**
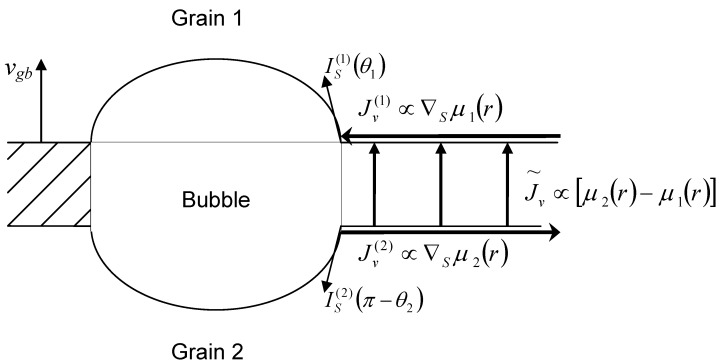
Schematic representation of vacancy fluxes along and across the grain boundary.

As illustrated in Figure 4, the chemical potential gradients along the grain face surfaces, $\nabla_{s}\mu_{1}$ and $\nabla_{s}\mu_{2}$, determine the vacancy surface fluxes $J_{v}^{\left(1\right)}$ and $J_{v}^{\left(2\right)}$, introduced in Equation (49), whereas the chemical potential drop across the grain boundary $\delta \mu (r)=\mu_{2}(r)-\mu_{1}(r)$ determines the vacancy flux across the grain boundary:

(59) $${\tilde{J}}_{v}(r)=u_{gb}\delta \mu (r)/\Omega^{2}$$

Integrating this flux over the grain boundary area unoccupied with bubbles and using Equation (57) one can calculate the grain boundary velocity:

(60) $$v_{gb}=\frac{u_{gb}}{\Omega \pi R_{c}^{2}}\int_{\rho_{b}}^{R_{c}}(\mu_{2}(r)-\mu_{1}(r))2\pi rdr=u_{gb}n_{b}[(\sigma_{2}-\sigma_{1})\pi R_{c}^{2}-(\frac{2\gamma_{s}}{R_{2}}-\frac{2\gamma_{s}}{R_{1}})\pi \rho_{b}^{2}]$$

The second term on the r.h.s. of Equation (60) is calculated from Equation (48):

(61) $$\frac{2\gamma_{s}}{R_{2}}-\frac{2\gamma_{s}}{R_{1}}=2\varepsilon +\Delta G$$

Substitution of Equations (58) and (61) in Equation (60) results in:

(62) $$v_{gb}=u_{gb}\lfloor \Delta G(1-n_{b}\cdot \pi \rho_{b}^{2})-2\varepsilon n_{b}\cdot \pi \rho_{b}^{2}\rfloor$$

which exactly coincides with Equation (51). 

An additional relationship between $v_{gb}$ and ε can be obtained from the balance equation, Equation (49), if the surface vacancy fluxes $J_{v}^{\left(1\right)}$ and $J_{v}^{\left(2\right)}$ are properly ascertained. These fluxes obey the continuity equations on each face of the grain boundary, which in the system of coordinates moving along with the grain boundary take the form:

(63) $${\vec{\nabla }}_{s}\cdot {\tilde{J}}_{v}^{\left(1,2\right)}\pm {\tilde{J}}_{v}(r)\mp v_{gb}\Omega^{-1}=0$$

or

(64) $$\frac{D_{gb}w\Omega }{kT}\nabla_{s}^{2}\mu_{1,2}\pm \frac{u_{gb}(\mu_{2}-\mu_{1})}{\Omega }\mp v_{gb}=0$$

with the boundary conditions:

(65) $${\left(\frac{d\mu_{1,2}}{dr}\right)}_{r=R_{c}}=0,\;\mathrm{and}\;\mu_{1,2}(\rho_{b})=(\frac{2\gamma_{s}}{R_{1,2}}-P_{b})\Omega$$

It is straightforward to see that integration of Equation (64) over the surface non-occupied with the bubbles, directly results in the first part of Equation (60), if Equation (49) is valid.

Superposition of equations (64) forms the system of Laplace and Helmholtz type equations for $\mu_{2}+\mu_{1}$ and $\mu_{2}-\mu_{1}$ with the solution obeying Equation (65), which determines the vacancy fluxes at the bubble surface [15]:

(66) $$J_{v}^{\left(1\right)}=-J_{v}^{\left(2\right)}=\frac{D_{gb}w\Omega }{2kT}\left(\Delta G+2\varepsilon -\frac{v_{gb}}{u_{gb}}\right)\chi \frac{K_{1}(\chi \rho_{b})I_{1}(\chi R_{c})-I_{1}(\chi \rho_{b})K_{1}(\chi R_{c})}{I_{0}(\chi \rho_{b})K_{1}(\chi R_{c})+K_{0}(\chi \rho_{b})I_{1}(\chi R_{c})}$$

where $\chi =\sqrt{2\mu_{gb}kT/D_{gb}w\Omega }$, and $I_{0,1}(x)$ and $K_{0,1}(x)$ represent the first and the second modified Bessel functions of the zeroth and first kind, respectively.

Substitution of Equation (66) in Equation (49) results in the additional relationship for the grain boundary velocity:

(67) $$v_{gb}=(\Delta G+2\varepsilon )\pi \rho_{b}^{2}\left[\frac{D_{gb}w\Omega \chi \phi (\chi \rho_{b},\chi R_{c})}{\pi kT\rho_{b}^{3}}{\left(1+\frac{D_{gb}w\Omega \chi \phi (\chi \rho_{b},\chi R_{c})}{kT\rho_{b}u_{gb}}\right)}^{-1}\right]$$

where $\phi (\chi \rho_{b},\chi R_{c})=\frac{K_{1}(\chi \rho_{b})I_{1}(\chi R_{c})-I_{1}(\chi \rho_{b})K_{1}(\chi R_{c})}{I_{0}(\chi \rho_{b})K_{1}(\chi R_{c})+K_{0}(\chi \rho_{b})I_{1}(\chi R_{c})}$.

In the meaningful limit $R_{c}\gg w$, $\rho_{b}\geq w$, one has with a very good accuracy $\phi (\chi \rho_{b},\chi R_{c})\approx 1$, until $\rho_{b}<R_{c}$:

(68) $$v_{gb}=\left(\Delta G+2\varepsilon \right)\pi \rho_{b}^{2}\left[\frac{D_{gb}w\Omega \chi }{\pi kT\rho_{b}^{3}}{\left(1+\frac{D_{gb}w\Omega \chi }{kT\rho_{b}u_{gb}}\right)}^{-1}\right]=F_{b}b_{f}$$

Superposition of Equations (62) and (68) allows exclusion of the parameter *ε* and final calculation of the grain boundary velocity:

(69) $$v_{gb}=\frac{u_{gb}b_{f}/n_{b}}{u_{gb}+b_{f}/n_{b}}\Delta G$$

where the bubble mobility is presented by the expression in brackets of Equation (68):

(70) $$b_{f}=\frac{D_{gb}w\Omega \chi }{\pi kT\rho_{b}^{3}}{\left(1+\frac{D_{gb}w\Omega \chi }{kT\rho_{b}u_{gb}}\right)}^{-1}$$

A further simplification of Equation (70) can be attained using evaluation of the grain boundary mobility $u_{gb}$ in Equation (53) resulting in $\chi \approx w^{-1}$. In this case the bubble mobility can be approximated as:

(71) $$b_{f}=\frac{D_{gb}\Omega }{\pi kT\rho_{b}^{3}}{\left(1+\frac{2w}{\rho_{b}}\right)}^{-1}\approx \frac{D_{gb}\Omega }{\pi kT\rho_{b}^{3}}$$

For gas bubbles in UO_2_ the surface diffusion mechanism of bubble migration generally dominates over the two other mechanisms of lattice diffusion and gas phase transport (see, e.g. [29]). For this reason, in derivation of Equations (70) and (71) it was implicitly assumed that the surface diffusion of uranium atoms along the two segments (upper and lower) of the bubble surface disconnected by the grain boundary, was fast enough to redistribute in the bubble all vacancies absorbed from the upper surface of the grain boundary (flux $J_{v}^{\left(1\right)}$) and desorbed to the lower one (flux $J_{v}^{\left(2\right)}$), in order to sustain its steady-state lenticular shape in the course of grain boundary migration. This assumption can be explicitly grounded if one compares two expressions for the bubble mobility by the new mechanism, Equation (71), and by the bubble surface diffusion mechanism. For the lenticular grain face bubble the mobility by the surface diffusion mechanism was derived in [15]:

(72) $$u_{s}\approx \frac{3D_{s}w\Omega }{2\pi kT\rho_{b}^{4}}\frac{\sin^{4}\theta_{0}}{1-\cos^{3}\theta_{0}}\approx \frac{3D_{s}w\Omega }{4\pi kT\rho_{b}^{4}}$$

where *θ*_0_ ≈ *θ*_1_ ≈ *θ*_2_ ≈ 50° for UO_2_.

Therefore, comparing Equations (71) and (72) one can see that $u_{b}/u_{s}\approx \left(D_{gb}/D_{s}\right)\left(\rho_{b}/w\right)$. From analysis of experimental data for UO_2_ (see below Section 6) one can conclude that $D_{s}$ exceeds $D_{gb}$ by 1–2 orders of magnitude in a wide range of temperatures above 1000 K, increasing with temperature. At higher temperatures *T* ≈ 2000 K, when the grain growth becomes noticeable, the ratio $D_{s}/D_{gb}$ attains 3 orders of magnitude. Therefore, for the practical interval of bubble sizes $w\leq \rho_{b}\leq 10^{3}w$, *i.e.*, from ≈ 1 nm up to ≈ 1 μm, the ratio $u_{b}/u_{s}$ is still small.

## 4. Retarding Effect of Peripheral Bubbles on Grain Growth in Irradiated Fuel

In the current section the new mechanism of the peripheral (edge and corner) intergranular bubble migration associated with vacancy fluxes along grain boundary is considered following the original publication of the author [23].

**Figure 5:**
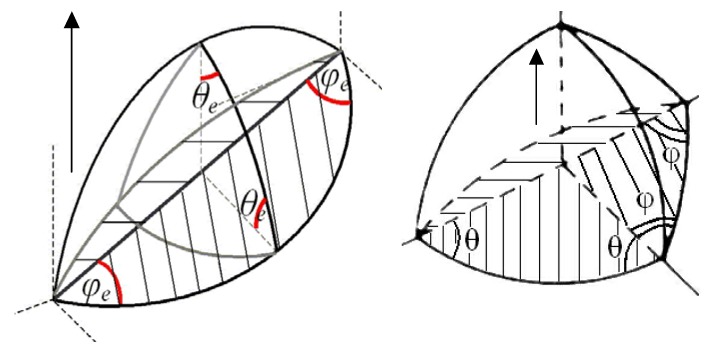
Peripheral grain boundary bubbles (edge and corner).

The shape of UO_2_ grains is considered as a truncated octahedron or tetrakaidecahedron (TDK) [30]. The TDK has 14 faces, six of which are square and eight hexagonal, 36 edges and 24 corners. When packed together an array of TDKs can fill all available space in a solid and thus represents an appropriate basic building block. The meeting point of each grain face is shared by two grains, each grain edge by three grains and each grain corner by four grains. Face bubbles are uniformly distributed over these faces with the surface concentration $n_{f}=1/\pi R_{c}^{2}$ and bubbles of the two other types (*N_e_* edge and *N_c_* corner bubbles, associated with one grain face) are located on the periphery of the faces, Figure 5. 

**Figure 6:**
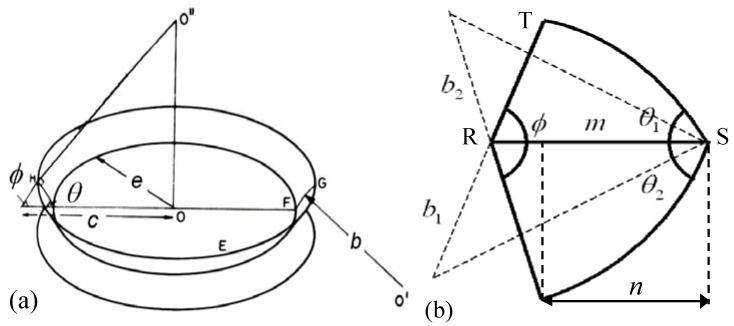
**(a)** Toroid formation. **(b)** Toroid cross-section.

Tucker has further rationalized the TDK structure by assuming that the grain is composed of fourteen circular faces with radius *e* [31]. The grain edge porosity is represented in this model by a tube (or “toroid”) threading around the circumference of the grain face. The toroid is formed by rotation of arc GH (IH) around the vertical axis passing through the centre of grain face, as shown in Figure 6. The volume of the toroid is equal to the volume of edge porosity *V_∑_* associated with one grain (see Appendix A).

### 4.1. Continuity Equation for Vacancy Fluxes on Grain Face

The vacancy flux to the periphery of the grain face will be calculated within the mean field approximation. In this approach vacancy sinks into the face bubbles are represented as a sink uniformly distributed over the grain face with the strength:

(73) $$S=\frac{J_{1,2}^{face}(r=\rho_{b})\cdot 2\pi \rho_{b}\Omega }{\pi {R_{c}}^{2}}$$

From the condition of coherent relocation of the grain boundary-bubble complex, Equation (49), the distributed sink strength (73) of face bubbles occupying the grain face with the surface concentration $n_{f}=1/\pi R_{c}^{2}$ takes the form:

(74) $$S=\mp \rho_{b}^{2}v_{gb}/R_{c}^{2}$$

The continuity equations at each face of the grain boundary in the system of coordinates moving along with the grain boundary, generalises Equation (64) and takes the form:

(75) $$-{\vec{\nabla }}_{S}J_{1,2}\pm u_{gb}\left(\mu_{2}-\mu_{1}\right)/\Omega \mp v_{gb}\mp S=0$$

with the boundary conditions at the grain face periphery [31]:

(76) $$\mu_{1,2}\left(r=e\right)=\left(K_{1,2}\gamma_{s}-p_{tor}\right)\Omega$$

where $K_{1,2}$ is the toroid surface curvature and $p_{tor}$ is the gas pressure in the toroid. The first term in Equation (75) is the vacancy flux along the upper (lower) face of the grain boundary:

(77) $$J_{1,2}=-\frac{D_{gb}w}{kT\Omega }\nabla_{S}\mu_{1,2}$$

and the second term is the vacancy flux across the grain boundary.

Superposition of equations (75) forms the system of Laplace and Helmholtz type equations for $\mu_{2}+\mu_{1}$ and $\mu_{2}-\mu_{1}$ which has solution (see Appendix B):

(78) $$\mu_{1,2}(r)=-\frac{\Delta G}{2}\mp \left(\left(\Delta G+2\varepsilon_{tor}\right)\Omega -\left(v_{gb}+S\right)\frac{\Omega }{u_{gb}}\right)\frac{I_{0}\left(\chi r\right)}{2I_{0}\left(\chi e\right)}\mp \left(v_{gb}+S\right)\frac{\Omega }{2u_{gb}}$$

where $\varepsilon_{tor}=K_{2}\gamma_{S}-p_{tor}=p_{tor}-K_{1}\gamma_{S}-\Delta G$ is the deviation parameter (similar to that introduced for the face bubbles in Equation (48)).

Correspondingly, the total vacancy flux to (from) the grain boundary periphery with perimeter 2*πe*, along the upper (lower) surface of the grain boundary is:

(79) $$J_{1,2}=\mp \left(\left(\Delta G+2\varepsilon_{tor}\right)-\frac{v_{gb}+S}{u_{gb}}\right)\frac{\pi eD_{gb}w\chi }{kT}\frac{I_{1}\left(\chi e\right)}{I_{0}\left(\chi e\right)}$$

Self-consistency of the above presented mean-field approximation is shown in Appendix C.

### 4.2. Coherent Migration of Edge and Corner Bubbles

The calculated vacancy flux to the periphery should be distributed between two kinds (edge and corner) of the peripheral bubbles. Such a distribution can be described by two weighing factors $\eta_{e},\eta_{c}$, so that the vacancy flux to (from) each kind of the bubbles is equal to:

(80) $$I_{1,2}^{\left(e,c\right)}=\eta_{e,c}J_{1,2}/N_{e,c}$$

These factors can be found from condition of the equal velocities of the two kinds of the bubbles:

(81) $$v_{e}=v_{c}=v_{gb}$$

Indeed, displacement of the grain boundary with the attached peripheral bubbles is associated with relocation of the bubbles cross-sections, dashed in Figure 5. Areas of these cross-sections are:

(82) $$S_{e}=2R_{e}^{2}\left(\varphi_{e}-\cos\varphi_{e}\sin\varphi_{e}\right)$$

(83) $$S_{c}=2R_{c}^{2}\left(\theta_{c}-\frac{\pi }{6}-\frac{\sin(\theta_{c}-\pi /6)}{\sin2\pi /3}\cos\theta_{c}\right)+R_{c}^{2}\left(\varphi_{c}-\pi /4-\frac{\sin(\theta_{c}-\pi /6)}{\sin2\pi /3}\cos\varphi_{c}\right)$$

Therefore, a part of the bubble volume swept by the moving grain boundary $dV_{e,c}=S_{e,c}v_{gb}$ should be compensated by the vacancy fluxes $I_{1,2}^{\left(e,c\right)}$ over the grain boundary faces, which provide bubbles migration with the grain boundary velocity $v_{gb}$ (compare with a similar consideration for the face bubbles in Equation (49)):

(84) $$v_{gb}S_{e,c}=I_{1,2}^{\left(e,c\right)}\Omega$$

Comparing Equations (80) and (84) one can evaluate:

(85) $$\eta_{e,c}=\frac{S_{e,c}N_{e,c}}{S_{c}N_{c}+S_{e}N_{e}}$$

After substitution of Equations (79) and (80) in Equation (84) one obtains:

(86) $$v_{gb}=\frac{D_{gb}\Omega }{kT}\left(\Delta G+2\varepsilon_{tor}\right){\left[\frac{S_{e}N_{e}}{\pi e\eta_{e}}+2w\left(1+\frac{\rho_{b}^{2}}{R_{c}^{2}}\right)\right]}^{-1}$$

### 4.3. Forces Exerted on Peripheral Bubbles and Toroid

In order to calculate the velocity of the grain boundary with intergranular bubbles one should calculate forces, exerted on the peripheral bubbles by the boundary. As shown in Section 3.2, the force acting on the face bubble is:

(87) $$F_{f}=\left(\Delta G+2\varepsilon_{f}\right)\pi \rho_{b}^{2}=2\gamma_{S}\left(1/R_{f2}-1/R_{f1}\right)\pi \rho_{b}^{2}$$

where $\varepsilon_{f}=2\gamma_{S}/R_{f2}-p_{f}$, from the condition of the coherent migration of the grain boundary and the attached bubble.

Similarly to consideration of face bubbles in Section 3.2, the integral of the normal stress over the unoccupied area associated with one «toroidal bubble» must equal the total load applied to each face of the grain boundary. Hence, one obtains:

(88) $$\Omega^{-1}\int_{S_{face}}\mu_{1,2}\left(r\right)2\pi rdr=\sigma_{1,2}\pi e^{2}-\left(K_{1,2}\gamma_{s}-p_{tor}\right)S_{tor}$$

where the first term on the r.h.s. arises from the normal stresses $\sigma_{1,2}$ at each of two surfaces of the grain boundary in the absence of the attached toroidal bubble. In the presently considered case of the moving boundary these stresses uphold the pressure gradient Δ*G* across the grain boundary, *i.e.*, $\sigma_{2}=\sigma_{1}+\Delta G$ (see Equation (58)).

The second term on the r.h.s. of Equation (88) expresses the force which the toroid surface tension exerts on the boundary. This term can be calculated, for example, analogously to the l.h.s. of Equation (57) as the integral of the normal stress on the toroidal bubble, $\mu_{1,2}\left(R_{1,2},\theta \right)=\sigma_{nn}^{\left(1,2\right)}\left(\theta \right)\Omega =\left(K_{1,2}\gamma_{s}-p_{tor}\right)\Omega$, over the corresponding segment of the toroidal bubble:

(89) $$F_{1,2}=\int \sigma_{nn}^{\left(1,2\right)}\cos\theta \cdot dS_{1,2}=\left(K_{1,2}\gamma_{s}-p_{tor}\right)S_{tor}$$

where $S_{tor}$ is the toroid projection area, calculated in Appendix A.

In order to sustain a coherent migration of the grain boundary and the attached toroid, the vacancy fluxes $J_{v}^{\left(1\right)}$ and $J_{v}^{\left(2\right)}$, which are determined by the pressure differences $\Delta p_{1}$ and $\Delta p_{2}$, respectively, should obey the relationship $J_{v}^{\left(1\right)}=-J_{v}^{\left(2\right)}$, which results in the condition $\Delta p_{1}=-\Delta p_{2}$:

(90) $$p_{tor}-\Delta G-K_{1}\gamma_{S}+p_{tor}-K_{2}\gamma_{S}=0$$

Therefore, the force acting on the toroid $F_{tor}$ is equal to:

(91) $$F_{tor}=F_{2}-F_{1}=\left(K_{2}\gamma_{S}-K_{e}\gamma_{S}\right)S_{tor}=\left(\Delta G+2\varepsilon_{tor}\right)S_{tor}$$

where

(92) $$\varepsilon_{tor}=K_{2}\gamma_{S}-p_{tor}$$

Similarly one obtains the force acting on the edge and corner bubbles:

(93) $$F_{e,c}=\left(\Delta G+2\varepsilon_{e,c}\right)\cdot S_{e,c}$$

where $\varepsilon_{e,c}=\varepsilon_{e,c}\left(R_{e,c1},R_{e,c2},p_{e,c}\right)$ from the corresponding conditions for coherent migration of the boundary and the attached bubbles.

### 4.4. Grain Boundary Retarding Effect

The grain boundary velocity depends on the net force acting on the boundary:

(94) $$v_{gb}=\frac{dR_{gr}}{dt}=u_{gb}\left(\Delta G-F_{f}n_{f}-F_{e}n_{e}-F_{c}n_{c}\right)$$

where $n_{i}$ is the surface concentration of the *i*-th type bubbles.

Each grain face with the surface area *πe*^2^ has one toroid bubble, so, one should assign to the toroid bubbles the surface concentration 1/*πe*^2^. The force acting on the toroid must be equal to the sum of forces acting on the edge and corner bubbles:

(95) $$F_{tor}=\left(F_{e}n_{e}+F_{c}n_{c}\right)\pi e^{2}$$

In this case the grain boundary retarding effect associated with the toroid coincides with one associated with the peripheral bubbles. Therefore, the grain boundary velocity can be represented in the form:

(96) $$v_{gb}=u_{gb}\left(\Delta G-F_{f}n_{f}-F_{tor}/\pi e^{2}\right)$$

On other hand

(97) $$v_{gb}=F_{f}b_{f}$$

where $b_{f}=\frac{D_{gb}\Omega }{kT\pi \rho_{b}^{3}}$ as derived in Section 3.2, Equation (71). 

Substitution of Equation (91) in Equation (86) gives:

(98) $$v_{gb}=\frac{D_{gb}\Omega }{kTS_{tor}}{\left[\frac{S_{e}N_{e}}{\pi e\eta_{e}}+2w\left(1+\frac{\rho_{b}^{2}}{R_{c}^{2}}\right)\right]}^{-1}F_{tor}$$

Superposition of Equations (94)–(98) results in the relationship for the boundary velocity (see Appendix D):

(99) $$v_{gb}=u_{gb}\Delta G{\left[1+u_{gb}{\left(\frac{D_{gb}\Omega }{kT\pi \rho_{b}^{3}}\right)}^{-1}n_{f}+u_{gb}\left(\frac{S_{e}N_{e}}{\pi e\eta_{e}}+2w\left(1+\frac{\rho_{b}^{2}}{c^{2}}\right)\right)\cdot {\left(\frac{D_{gb}\Omega }{kT}\right)}^{-1}\frac{S_{tor}}{S_{face}}\right]}^{-1}$$

For a more realistic description of grain growth with consideration of size distribution and coalescence of grains using the same procedure as for derivation of Equation (14), one can obtain for the mean grain velocity a new relationship instead of Equation (99):

(100) $${\bar{v}}_{gb}=v_{gb}^{\left(0\right)}{\left[1+\frac{81v_{gb}^{\left(0\right)}{\bar{R}}_{gr}kT}{8D_{gb}\Omega \xi \gamma_{gb}}\left(\pi \rho_{b}^{3}n_{f}+\frac{S_{tor}}{S_{face}}\left(\frac{S_{e}N_{e}}{\pi e\eta_{e}}+2w\left(1+\frac{\rho_{b}^{2}}{R_{c}^{2}}\right)\right)\right)\right]}^{-1}$$

where $S_{tor}\approx 2\pi e^{2}\sqrt{\Delta V/V_{0}}$ (see Appendix A). 

### 4.5. Effect of Bubbles Coalescence during Grain Growth

Further improvement of the model concerns additional consideration of the intergranular bubbles coalescence owing to grains shrinkage. Similarly to consideration of (empty) pores coalescence by Kingery and François [26], one can assume that, as grains are removed in the growth process, bubbles migrating with the boundaries are brought together, and corner bubbles growth occurs together with the grain growth. This process results in the increased retarding effect exerted by corner bubbles.

In the standard approach all gas content swept by a moving boundary in a shrinking grain was uniformly distributed among various types of intergranular bubbles, characterized by invariable surface concentrations of these types. In the new approach the procedure of collecting all intragranular gas content into intergranular bubbles is conserved, however, additionally all intergranular gas content is collected in corner bubbles after complete grain disappearance. 

Correspondingly, the numbers of gas atoms in the intergranular bubbles $N_{f,e,c}$ are redistributed in the following way:

(101) $$dN_{f,e}/N_{f,e}=-dN_{gr}/N_{gr}=-3d{\bar{R}}_{gr}/{\bar{R}}_{gr}$$

(102) $$dN_{c}/\left(N_{f}+N_{e}\right)=3d{\bar{R}}_{gr}/{\bar{R}}_{gr}$$

where $N_{gr}$, ${\bar{R}}_{gr}$ are the number of grains and the mean grain radius at time *t*, respectively. 

The effect becomes essential when a manifold increase of grain size occurs, e.g. under low irradiation rates. 

## 5. Combined Retarding Effect of Pores and Bubbles on Grain Growth in Irradiated Fuel

As shown in Section 2, consideration of fuel densification (*i.e.*, porosity reduction) owing to thermal evaporation of vacancies from pores allows explanation of complicated grain growth kinetics characterised by non-integer growth exponents observed in the tests [20] on thermal annealing of porous UO_2_ fuel. However, under irradiation conditions pores additionally shrink owing to vacancy knockout mechanism [32,33] based on the assumption that fission fragments passing through a pore can, via atomic collisions, effectively knock vacancies from the pore, therefore, both pore shrinkage mechanisms should be considered simultaneously. 

On the other hand, intergranular gas bubbles grow up under irradiation conditions owing to sinking of fission gas to the grain boundaries leading to significant fuel swelling. Therefore, the retarding effect on the moving grain boundaries from both types of intergranular porosity, *i.e.*, pores and gas-filled bubbles, should be simultaneously considered in the general case of porous fuel under irradiation conditions.

The additional mechanism of the irradiation-induced vacancy knockout from pores [32,33] results in the following rate equation (instead of Equation (33)):

(103) $$\frac{d{\bar{R}}_{p}}{dt}=\frac{{\bar{R}}_{p}}{{\bar{R}}_{gr}}\frac{d{\bar{R}}_{gr}}{dt}-\frac{\alpha }{3{\bar{R}}_{p}^{3}}-2\lambda \eta \Omega F$$

where the new term in the r.h.s. corresponds to the vacancy knockout by fission fragments:

(104) $$\frac{\partial {\bar{V}}_{p}}{\partial t}=-8\pi \lambda \eta \Omega F{\bar{R}}_{p}^{2}$$

*F* is a fission rate, *η* = 100 is the number of vacancies knocked out of a pore per collision with the fission fragment and *λ* = 10^–6^ m is the length of the fission fragment path. 

In Hillert’s mean field approximation for grains coalescence, Equation (17), the mean grain boundary velocity supplemented with the additional type of inclusions (pores) takes the form:

(105) $${\bar{v}}_{gb}=\frac{d{\bar{R}}_{gr}}{dt}=v_{gb}^{\left(0\right)}{\left(1+\frac{81v_{gb}^{\left(0\right)}{\bar{R}}_{gr}}{8\xi \gamma_{gb}}\left(n_{f}b_{f}^{-1}+n_{e}b_{e}^{-1}+n_{c}b_{c}^{-1}+n_{p}b_{p}^{-1}\right)\right)}^{-1}$$

where, for the UO_2_ typical case of relatively large pores with *R_p_* ≥ 1 μm the surface diffusion mechanism controls the pore migration kinetics:

(106) $$b_{p}\approx \frac{3D_{s}\Omega^{4/3}}{2\pi kTR_{p}^{4}}$$

Finally, taking into consideration the additional term associated with the pores, Equation (100) takes the form:

(107) $${\bar{v}}_{gb}=v_{gb}^{\left(0\right)}{\left[1+\frac{81v_{gb}^{\left(0\right)}{\bar{R}}_{gr}}{8\xi \gamma_{gb}}\left(n_{f}b_{f}^{-1}+\frac{S_{tor}}{S_{face}}{\left(\frac{D_{gb}\Omega }{kT}\right)}^{-1}\left(\frac{S_{e}N_{e}}{\pi e\eta_{e}}+2w\left(1+\frac{\rho_{b}^{2}}{R_{c}^{2}}\right)\right)+n_{p}b_{p}^{-1}\right)\right]}^{-1}$$

which in combination with Equation (103) determines the grain growth kinetics controlled by pore and bubble migration in irradiated fuel.

## 6. Model Implementation and Validation

New model was implemented in the integral code MFPR, which is developed for analysis of UO_2_ fuel microstructure evolution and fission products release under irradiation conditions [24,25]. In particular, the code simulates growth and coalescence of intergranular bubbles, and thus the newly implemented model allows self-consistent calculation of the grain boundary retarding effect by the attached bubbles. 

The normal grain growth kinetics in non-irradiated and non-porous fuel is represented in the MFPR code in the standard parabolic form:

(108) $$v_{gb}^{\left(0\right)}=v_{0}\left({\bar{R}}_{0}/{\bar{R}}_{gr}\right)\cdot \exp\left(-E_{gb}/T\right)$$

with activation energy *E_gb_* = 44200 K recommended by Speight and Greenwood [13]. Correspondingly, the diffusivity across the grain boundary evaluated from Equation (53) following Burke and Turnbull [9] is $D_{gb}^{\left(p\right)}$ ≈ 4 × 10^-6^∙exp(–44200/T) m^2^/s, whereas the diffusivity along the grain boundary measured by Alcock et al. [34] is $D_{gb}^{\left(l\right)}$ ≈ 4 × 10^-6^∙exp(–35250/T) m^2^/s. This disagreement can be explained by an assumption that the diffusivity along the grain boundary ($D_{gb}^{\left(l\right)}$) differs from the diffusivity across the grain boundary ($D_{gb}^{\left(p\right)}$) [15]. 

Under this assumption Equation (70) takes the form $u_{b}=D_{gb}^{\left(l\right)}w\Omega \chi /\pi kT\rho_{b}^{3}$, where $\chi =\sqrt{2u_{gb}kT/D_{gb}^{\left(l\right)}w\Omega }$ and $u_{gb}\approx D_{gb}^{\left(p\right)}\Omega /2wkT$, *i.e.*, $\chi =w^{-1}\sqrt{D_{gb}^{\left(p\right)}/D_{gb}^{\left(l\right)}}$. Therefore, instead of Equation (71) one gets a modified expression for the bubble mobility:

(109) $$u_{b}\approx \frac{\Omega }{\pi kT\rho_{b}^{3}}\sqrt{D_{gb}^{\left(p\right)}D_{gb}^{\left(l\right)}}$$

and instead of Equation (107) one gets a modified expression for grain boundary velocity:

(110) $${\bar{v}}_{gb}=v_{gb}^{\left(0\right)}{\left[1+\frac{81v_{gb}^{\left(0\right)}{\bar{R}}_{gr}kT}{8\sqrt{D_{gb}^{\left(p\right)}D_{gb}^{\left(l\right)}}\Omega \xi \gamma_{gb}}\left(\pi \rho_{b}^{3}n_{f}+\sqrt{D_{gb}^{\left(p\right)}D_{gb}^{\left(l\right)}}\Omega n_{p}b_{p}^{-1}+\frac{S_{tor}}{S_{face}}\left(\frac{S_{e}N_{e}}{\pi e\eta_{e}}+2w\left(1+\frac{\rho_{b}^{2}}{R_{c}^{2}}\right)\right)\right)\right]}^{-1}$$

### 6.1. Turnbull’s Tests

Validation of the modified code version was performed against Turnbull’s tests [8], where high-temperature grain growth in irradiated UO_2_ fuel was measured. In these experiments the effect of grain size on the swelling and gas release properties of uranium dioxide was studied. Small cylindrical specimens 10 mm long and 3 mm diameter were prepared from 2% enriched uranium dioxide of near theoretical density. The fuel samples were irradiated at *T* = 1750 °C for period of 2, 4 and 6 months in the UKAEA reactor DIDO in a flux of ≈ 2.4 × 10^17^ thermal neutrons/m^2^∙s. During irradiation the temperature was maintained by electrical heating; fission heating produced a temperature gradient within the specimens ≈ 100 °C from centre to surface. There were three types of samples with the initial grain diameter *d_gr_* = 7 μm (specimens A and B) and 40 μm (specimen C), the latter being produced by preliminary annealing of specimens A during 72 hours at *T* = 1700 °C in hydrogen. Specimens B and C were pre-irradiated to 0.02% burn-up at 80 °C. So, the following identification of the specimens is used:

- specimen A, 7 μm starting grain size;
- specimen B, 7 μm starting grain size, pre-irradiated to 0.02% burn-up at 80 °C;
- specimen C, 40 μm starting grain size, pre-irradiated to 0.02% burn-up at 80 °C.

Examination of large-grained specimen C showed the unchanged average grain size, whereas specimens A and B exhibited identical grain growth characteristics with the grain size increasing from 7 μm to 18 μm after 6 months irradiation. 

The density of the samples was close to theoretical one, for this reason, parameters of Equation (108) were fitted to reproduce the out-of-pile annealing behaviour of specimen C (*i.e.*, growing from 7 to 40 µm during 72 hours at *T* = 1700 °C): $2{\bar{R}}_{0}$ = 7 μm, *v*_0_ = 1.4 m/s . The surface concentration of grain face bubbles was estimated as ≈ 4 × 10^10^ m^-2^ from the post-test fracture surface image presented in [8].

**Figure 7:**
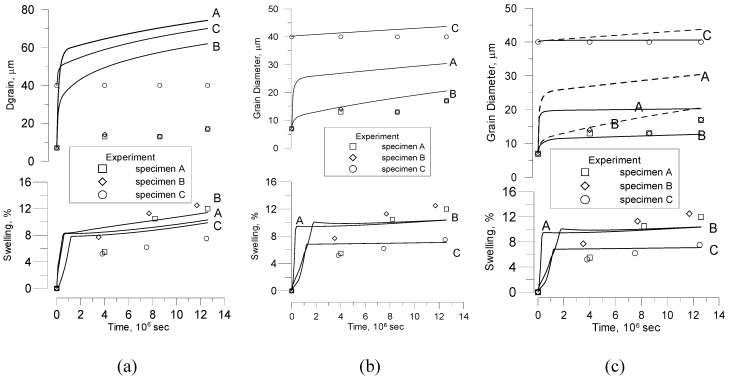
MFPR simulation of grain size in Turnbull’s test. **(a)** Standard migration mechanism. **(b)** Grain boundary diffusion mechanism applied to grain face bubbles. **(c)** Grain boundary diffusion mechanism applied to all grain boundary bubbles (*solid lines*) in comparison with case (b) (*dashed lines*).

In order to reveal retarding effect of grain face and peripheral bubbles, experimental results for grain growth kinetics and swelling are compared in Figure 7 with theoretical curves calculated with several versions of the MFPR code:

- The initial code version, where the standard (surface diffusion) mechanism is applied to the intergranular bubble migration (Figure 7a) [24];
- The intermediate version, where the new (grain boundary diffusion) mechanism is applied to the grain face bubbles migration and the standard (surface diffusion) mechanism is applied to the peripheral bubbles (Figure 7b) (Section 3 of the current paper) [15];
- The final version, where the new mechanism is applied to migration of all intergranular bubbles (Figure 7c) (Section 4 of the current paper).

In the standard approach using bubble mobility *u_s_* determined by the bubble surface diffusion mechanism, Equation (72), calculations strongly overpredict the measured grain growth kinetics for all three specimens, Figure 7a. 

The new model predicts a rather good agreement for the samples B and C and slightly overpredicts the growth of the sample A. From comparison of curves in Figure (7b) and Figure (7c) it is seen, that contribution of the peripheral bubbles to the grain boundary retarding effect is essential under the Turnbull’s test conditions.

### 6.2. Tests of Bourgeois et al.

In the tests [20] already discussed in Section 2, the normal grain growth kinetics of fresh UO_2_ pellets with the relative density *ρ* (in % with respect to the theoretical density) annealed in dry hydrogen was studied. Grain sizes and changes in density were measured for two batches, T0 and T12. Initial grain sizes in T0 and T12 were 8.8 and 10.4 μm, respectively, and the initial pore size can be obtained from expression for intergranular porosity:

(111) $$\rho =\frac{V_{gr}}{V_{gr}+6V_{pore}}$$

**Figure 8:**
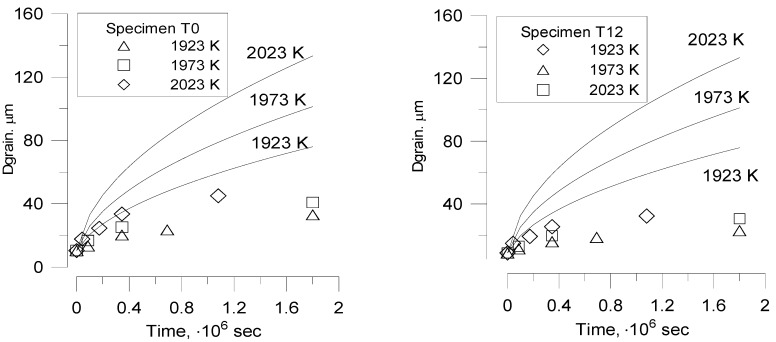
MFPR simulations of Bourgeois’ tests. Standard model for normal grain growth neglecting retarding effect of pores.

In order to reveal influence of pores on normal grain growth kinetics, at first calculations with the standard model, Equation (108), neglecting retarding effect of pores were performed, Figure 8.

**Figure 9:**
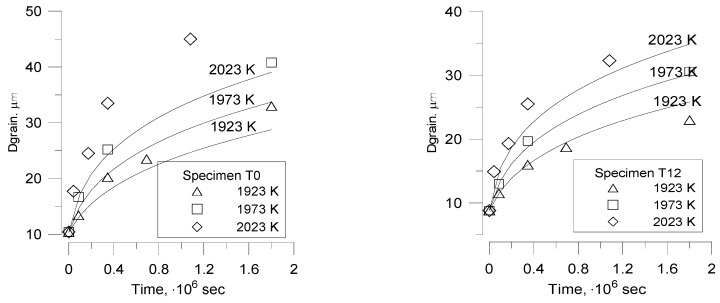
MFPR simulations of Bourgeois’ tests. New model for normal grain growth controlled by intergranular pores.

Results of calculations with the implemented new model are presented in Figure 9. Owing to a large uncertainty in determination of the diffusion coefficients and the coefficient *β* (from Equation (26)), these parameters were slightly adjusted in order to provide the best agreement between theoretical simulations and experimental points. In these simulations the grain boundary diffusion coefficient *D_gb_* was chosen as 4.2∙10^-6^∙exp(–35250/T) m^2^/s, and the surface diffusion coefficient *D_s_* as 56∙exp(–48945/T) m^2^/s, in a rather close agreement with estimations presented in [35]. As indicated in Section 2, the parameter β depends on the fuel porosity and for the typical fuel density 96-98% varies in the range 0.2–0.3; in calculations it was fixed as 0.15. Results of calculation with the improved code version show a reasonable agreement for the samples T12 and some underestimation of grain growth for the samples T0. 

**Figure 10:**
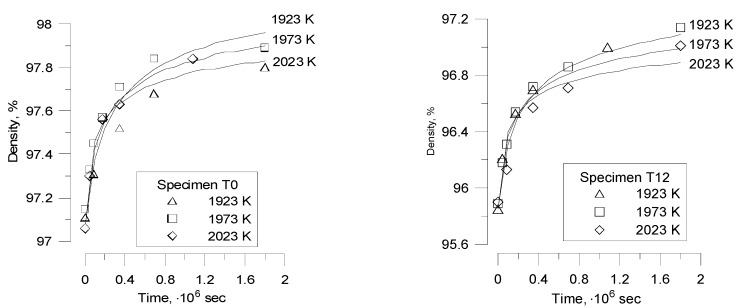
MFPR simulation of porosity evolution in Bourgeois’ tests.

### 6.3. Tests of MacEwan and Hayashi 

The MFPR code with the new advanced model was applied to simulation of the grain growth during post-irradiation annealing of uranium dioxide observed in the MacEwan and Hayashi tests [36]. In these tests the effect of prior exposure to irradiation at temperature below 400 °C on subsequent grain growth of UO_2_ samples with densities from 94 to 96% of theoretical density at 1800 °C during 24 hours was studied. Grain growth was reduced in all irradiated specimens, with nearly complete inhibition occurring by 4 × 10^19^ fissions/cm^3^. 

In order to reveal competitive influence of bubbles and pores on the grain growth kinetics in pre-irradiated UO_2_ samples, calculations were performed with two code versions, respectively including:

- The grain growth controlled only by bubbles migration (presented in Section 3 and Section 4);
- The grain growth controlled by migration of both intergranular pores and bubbles (final version presented in Sections 5).

The simulation results in comparison with the experimental data are presented in Table 1. It is evident from the simulation results that under irradiation exposure lower than 4 × 10^18^ fissions/cm^3^ the grain growth is limited mainly by intergranular pores, and for exposures greater than 4 × 10^18^ fissions/cm^3^ migration of intergranular bubbles becomes the rate controlling mechanism.

The grain growth kinetics exponents (presented in Table 1 in parentheses ulated curves using the least-squares method, differ from exponent *n* ≈ 2.5 measured in [36], however, they are in a reasonable agreement with the normal grain growth exponents, 3 < *n* < 4, measured in the tests of Bourgeois *et al*. [20]. 

**Table 1:** Simulation of the MacEwan and Hayashi tests.

Irradiation exposure, fiss./cm^3^	Final grain size, μm	Predicted grain size, μm (estimated value of grain growth exponent)	
		Grain growth controlled by bubbles	Grain growth controlled by bubbles and pores
0	16.0	38.5 (2.006)	16.75 (3.675)
3.8 × 10^15^	14.2	38.3 (2.013)	16.75 (3.626 )
3.6 × 10^16^	13.8	35 (2.225)	16.74 (3.671)
2.8 × 10^17^	10.8	21.9	16.3 (3.711)
4.4 × 10^18^	-	9.8	10.2
4.4 × 10^19^	8.2	7.2	7.2

## 7. Conclusions 

New mechanisms of the grain growth in irradiated and non-irradiated porous ceramic materials proposed and developed in the recent papers of the author, are reviewed in the current paper. As the first step of the new model development, Nichols’ approach [14] to consideration of the drag force exerted by attached bubbles and pores on migrating grain boundaries is combined with supplementary consideration of grains coalescence within Hillert’s mean field approach [11]. It is shown that the boundary migration rate becomes controlled by the movement of the second-phase particles with significantly smaller sizes than predicted in the simplified approach [14]. An additional consideration of various types of grain boundary pores and bubbles (*i.e.*, grain face, edge and corner) which exert different drag forces owing to their different shapes and sizes, was performed. 

However, Nichols’ analysis [14] is based on consideration of retarding effect of bubbles on moving boundary using the standard (lattice diffusion, gas phase transport or surface diffusion) mechanisms of bubble mobility derived for intragranular bubbles. This approach was re-considered in the present paper taking into account a more complicated, lenticular shape of the grain face bubbles. Furthermore, migration mechanism of the grain face bubbles might be essentially different from the intragranular bubbles, owing to their specific location on and interaction with a grain boundary. The new mechanism of the lenticular grain face bubble migration is associated with vacancy fluxes over the grain boundary surfaces to the bubble, which afford coherent relocation of the grain boundary-bubble complex. The calculated mobility of the grain face bubble is characterised by a slower dependence on its projected radius, $\propto \rho_{b}^{-3}$, in comparison with the surface diffusion mechanism, $\propto \rho_{b}^{-4}$, which sustains its steady-state lenticular shape in the course of bubble migration. In particular, for UO_2_ the new mechanism becomes the rate controlling step for bubbles migration in a wide range of their radii from ~ 1 nm to ~ 1 μm, and correspondingly, determines the drag force exerted by bubbles on the grain boundary.

The new mechanism of the grain boundary bubbles migration which controls the bubble mobility and determines the drag force exerted on the grain boundary, is further developed in application to the peripheral (edge and corner) intergranular bubbles. As a result, the growth kinetics of grains with different types of intergranular bubbles is calculated. It is shown that contribution of the peripheral bubbles to the retarding effect can be significant, especially under irradiation conditions with high fission rates in UO_2_ fuel.

The new model for the grain growth was also applied to consideration of as-fabricated porous fuel on the base of self-consistent simulation of grain growth and fuel densification, which occurs under annealing conditions owing to thermal evaporation of vacancies from pores. Under irradiation conditions the pore shrinkage is significantly increased owing to vacancy knockout from pores by fission particles, on the one hand, and intergranular bubbles growth takes place owing to sinking of fission gas atoms from grains, on the other hand. Simultaneous consideration of the intergranular bubbles and pores evolution allows further improvement of the model predictions for the grain growth under irradiation conditions. 

The new model was implemented in the integral code MFPR and validated against various test under annealing [8,20] and irradiation [36] conditions with various types (dense and porous) fuel pellets, with and without pre-irradiation. The new code predictions for these tests are essentially improved and are in a satisfactory agreement with observations. 

## Figures and Tables

**Figure 11:**
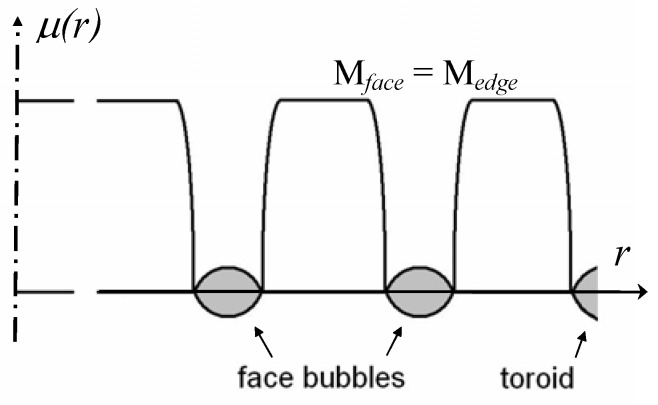
Chemical potential distribution.

## Appendix A. Toroid Geometry

The toroid is formed by rotation of arc GH (IH) around the vertical axis passing through the centre of grain face [31], as shown in Figure 6.

In the process of the grain boundary migration, the toroid changes its form similarly to grain face bubbles and one should distinguish between upper and lower surfaces of the toroid. Each of these surfaces is determined by their own radius of curvature *b*_1,2_ and contacted angle *θ*_1,2_, which obey the so called “lacing equation”:

(112) $$\frac{b_{1}\sin\left(\theta_{1}+\phi -\pi /2\right)}{\sin(\pi -\phi )}=\frac{b_{2}\sin\left(\theta_{2}+\phi -\pi /2\right)}{\sin(\pi -\phi )}(=m)$$

that is analogous to the relationship $\rho_{b}=R_{1,2}\sin\theta_{1,2}$ for the face bubbles. Equation (112) is deduced from «the useful relationship » presented in [31]:

(113) e=c+b⋅cos(θ+ϕ)⋅csc(ϕ)

The volume of the toroid is equal to the volume of edge porosity *V_Σ_* associated with one grain. Since edge bubbles are shared by three faces of grain and corner bubbles by four faces, the volume of the toroid is equal to the total sum of bubble volumes associated with one grain:

(114) $$0.5\cdot \left(V_{tor}^{up}+V_{tor}^{dwn}\right)=N_{e}V_{e}+N_{c}V_{c}=V_{\Sigma }$$

where $V_{tor}^{up,dwn}=V_{t}\left(b_{1,2},c_{1,2},\theta_{1,2},\phi_{1,2}\right)$ is the volume of the upper and lower parts of toroid. Approximate expression for fractional swelling was presented by Tucker [31] after substituting values *θ*_0_ ≈ 50º, $\phi_{0}\approx \arcsin6/7$ in the following form:

(115) $$\frac{\Delta V}{V_{0}}=\frac{V_{t}}{V_{cone}-V_{t}}={\left[{\left(0.5104r^{2}-0.0799r^{3}\right)}^{-1}-1\right]}^{-1}$$

where *Vcone* is the volume of grain cone, associated with one toroid, r=0.5557⋅b/c. The approximation Equation (115) is valid up to $\Delta V/V_{0}\leq 1$. For small $\Delta V/V_{0}<0.1$ Equation (115) can be simplified:

(116) $$\frac{\Delta V}{V_{0}}={\left[{\left(0.5104r^{2}\right)}^{-1}-1\right]}^{-1}$$

Since edge swelling normally does not exceed 0.1, one could obtain from Equation (116):

(117) $$b/c=2.52\sqrt{\Delta V/\left(V_{0}+\Delta V\right)}\approx 2.52\sqrt{\Delta V/V_{0}}$$

Substitution of Equation (117) in Equation (113) yields:

(118) $$c=e{\left(1-\sqrt{\Delta V/V_{0}}\right)}^{-1}\approx e\left(1+\sqrt{\Delta V/V_{0}}\right)$$

The area of toroid cutting off grain face is equal to:

(119) $$S_{tor}=\int_{S_{tor}}\cos\theta \cdot dS$$

where the integral in Equation (119) is taken over the toroid surface, resulting in:

(120) $$S_{tor}=\pi c^{2}-\pi e^{2}$$

From Equations (118) and (120) one can calculate *S_tor_* in the linear approximation at $\Delta V/V_{0}<<1$:

(121) $$S_{tor}\approx 2\pi e^{2}\sqrt{\Delta V/V_{0}}$$

The toroid surface curvature *K* at the point F in Figure 6a is equal to:

(122) $$K=\frac{1}{b}-\frac{\sin\theta }{c+b(\cos\theta \cot\phi -\sin\theta )}$$

The balance between the surface tension forces in the plane of the grain boundary has the form:

(123) $$\cos\theta_{1}+\cos\theta_{2}=\gamma_{gb}/\gamma_{S}$$

which in the linear approximation when $\theta_{1}\approx \theta_{2}\approx \theta_{0}$ takes the usual form: $\theta_{0}=\arccos(\gamma_{gb}/2\gamma_{S})\approx 50^{\circ }$.

## Appendix B. Solution of the Continuity Equations

In this Appendix will be searched the solution of Equation (75):

(124) $$\frac{D_{gb}w}{kT}{\vec{\nabla }}_{S}^{2}\mu_{1,2}\pm u_{gb}\left(\mu_{2}-\mu_{1}\right)/\Omega \mp v_{gb}\mp S=0$$

with the boundary conditions, Equation (76):

(125) $$\mu_{1,2}(r=e)=\left(\gamma_{S}K_{1,2}-p_{f}\right)\Omega ,\;{\left(\frac{\partial \mu_{1,2}}{\partial r}\right)}_{r\to 0}=0$$

Superposition of Equations (124) and (125) yields:

(126) $$\begin{array}{l} \Delta \left(\mu_{1}+\mu_{2}\right)=0,\\ \frac{D_{gb}w}{kT}\Delta \left(\mu_{1}-\mu_{2}\right)-\frac{2u_{gb}}{\Omega }\left(\mu_{1}-\mu_{2}\right)-2v_{gb}-2S=0. \end{array}$$

System of equations (126) can be written in the following form:

(127) $$\begin{array}{l} \Delta \left(\mu_{1}+\mu_{2}\right)=0,\\ \Delta \eta -\chi^{2}\eta -B=0, \end{array}$$

with a new variable $\eta =\mu_{1}-\mu_{2}$, where $B=\frac{2kT}{D_{gb}w}\left(S+v_{gb}\right)$ and $\chi^{2}=\frac{2u_{gb}kT}{\Omega D_{gb}w}>0$.

After substitution $N=\chi^{2}\eta +B$ one obtains the Laplace and Helmholtz type equations for variables $\left(\mu_{1}+\mu_{2}\right)$ and N, respectively:

(128) $$\begin{array}{l} \Delta \left(\mu_{1}+\mu_{2}\right)=0,\\ \Delta N-\chi^{2}N=0, \end{array}$$

which have general solutions:

(129) $$\begin{array}{l} \mu_{1}(r)+\mu_{2}(r)=C_{3}+C_{4}\ln r,\\ N(r)=C_{1}I_{0}\left(\chi r\right)+C_{2}K_{0}\left(\chi r\right), \end{array}$$

where constants $C_{1,2,3,4}$ are found from the boundary conditions Equation (125).

The solution is searched in the area 0<r<e. Since ln(r→0)→−∞ and $K_{0}\left(r\to 0\right)\to +\infty$, this results in $C_{4}=0$ и $C_{2}=0$. After determination of the constants $C_{1,3}$ from the boundary conditions, the final solution takes the form:

(130) $$\mu_{1,2}(r)=\frac{\left(\mu_{e1}+\mu_{e2}\right)}{2}\mp \frac{\mu_{e2}-\mu_{e1}-\left(v_{gb}+S\right)\Omega /u_{gb}}{2I_{0}\left(\chi s\right)}I_{0}\left(\chi r\right)\mp \left(v_{gb}+S\right)\frac{\Omega }{2u_{gb}}$$

## Appendix C. Validity of the Mean-Field Approximation

In calculation of the vacancy flux to the grain face periphery, the distributed sink strength of face bubbles, Equation (74), was used in the mean field approximation. The validity of such consideration will be checked in this Appendix.

First of all one should check self-consistency of the sink strength calculations. For this purpose, the sink strength into a face bubble located in the centre of the infinite grain face with uniformly distributed face bubbles surrounding this central bubble, should be evaluated.

The solution of the continuity equations for this system of bubbles with the pre-determined distributed sink strength *S*:

(131) $$D_{gb}w{\vec{\nabla }}_{S}^{2}{\tilde{\mu }}_{1,2}/kT\pm u_{gb}\left({\tilde{\mu }}_{2}-{\tilde{\mu }}_{1}\right)/\Omega \mp v_{gb}\mp S=0$$

is similar to the general solution of Equations (75) and (76), presented in Appendix B, but has different boundary conditions:

(132) $${\tilde{\mu }}_{1,2}(r=\rho_{b})=\left(\frac{2\gamma_{S}}{R_{1,2}}-p_{f}\right)\Omega ,\;{\left(\frac{\partial {\tilde{\mu }}_{1,2}}{\partial r}\right)}_{r\to +\infty }=0$$

Hence the terms with *I_0_*(*χr*) in the general solution, Equation (129), must be excluded, since they become infinitely large as *r* → +∞. Therefore, the solution takes the form:

(133) $${\tilde{\mu }}_{1,2}(r)=\left(\frac{\gamma_{S}}{R_{1}}+\frac{\gamma_{S}}{R_{1}}-p_{f}\right)\Omega \pm \frac{K_{0}(\chi r)}{2K_{0}(\chi \rho_{b})}\left(\frac{2\gamma_{S}\Omega }{R_{1}}-\frac{2\gamma_{S}\Omega }{R_{2}}+\frac{v_{gb}\Omega }{u_{gb}}+\frac{S\cdot \Omega }{u_{gb}}\right)\mp \frac{v_{gb}\Omega }{2u_{gb}}\mp \frac{S\cdot \Omega }{2u_{gb}}$$

where *R*_1,2_ are the curvature radii of the two surface segments of the central bubble that obey the geometrical relationship:

(134) $$R_{1}\cdot \sin\theta_{1}=R_{2}\cdot \sin\theta_{2}=\rho_{b}$$

From this solution one obtains the vacancy flux to the central bubble:

(135) $${\tilde{J}}_{1,2}(r=\rho_{b})={\frac{D_{gb}w\nabla {\tilde{\mu }}_{1,2}(r)}{kT}|}_{r=\rho_{b}}=\frac{D_{gb}w\chi }{2kT}\left(\Delta G+2\varepsilon_{f}-\frac{v_{gb}}{u_{gb}}-\frac{S}{u_{gb}}\right)$$

where $\varepsilon_{f}=2\gamma_{S}/R_{2}-p_{f}$. From Equation (53) the boundary mobility is equal to:

(136) $$u_{gb}=\frac{D_{gb}\Omega }{2kTw}$$

therefore, $\chi =\sqrt{2u_{gb}kT/D_{gb}w\Omega }=w^{-1}$, and Equation (68) takes the form:

(137) $$v_{gb}=\left(\Delta G+2\varepsilon_{f}\right)\frac{D_{gb}\Omega }{kT\rho_{b}}$$

On the other hand, the distributed sink strength into face bubbles is determined by Equation (73) as:

(138) $$S=\frac{J_{1,2}(r=\rho_{b})\cdot 2\pi \rho_{b}\Omega }{\pi {R_{c}}^{2}}$$

Substituting Equation (135) in Equation (137) and taking into account Equation (138) one obtains the equation for the unknown value *S*:

(139) $$S≅\frac{D_{gb}\pi \rho_{b}\Omega }{kT\pi c^{2}u_{gb}}\left(\frac{v_{gb}u_{gb}}{\pi \rho_{b}^{2}b_{f}}-v_{gb}-S\right)$$

which has the solution (using Equation (136)):

(140) $$S=\frac{2w\pi \rho_{b}v_{gb}}{\pi R_{c}^{2}}\left(\frac{\rho_{b}}{2w}-1\right){\left(1+\frac{2w\pi \rho_{b}}{\pi R_{c}^{2}}\right)}^{-1}$$

that coincides with Equation (74) in the realistic limit $w<<\rho_{b}$:

(141) $$S\approx \rho_{b}^{2}v_{gb}/R_{c}^{2}$$

Therefore, self-consistency of the sink strength calculation is confirmed.

Secondly, the self-consistency of the chemical potential distribution calculations has to be checked. On the one hand, it is straightforward to see that the chemical potential distribution around a face bubble, Equation (133), steeply varies in a narrow vicinity $\approx \chi^{-1}\approx w$ of each face bubble and is practically constant in-between the bubbles. Taking into account Equation (48), this constant value is equal to:

(142) $$M_{face}^{\left(1,2\right)}=\left(\frac{2\gamma_{S}}{R_{1}}+\frac{2\gamma_{S}}{R_{1}}-2p_{f}\right)\Omega \mp \frac{v_{gb}\Omega }{2u_{gb}}\mp \frac{S\Omega }{2u_{gb}}=\left(-\Delta G\mp \frac{\left(v_{gb}+S\right)}{2u_{gb}}\right)\Omega$$

with except of the region near the face bubbles.

On the other hand, solution of the equation for the vacancy diffusion to the edge of the grain face, Equation (78), has a similar form with the constant value outside a narrow zone ($\approx \chi^{-1}\approx w$) around the peripheral toroid, taking into account Equation (90):

(143) $$M_{edge}^{\left(1,2\right)}=\left(K_{e1}\gamma_{S}+K_{e2}\gamma_{S}-2p_{tor}\right)\Omega \mp \left(v_{gb}+S\right)\frac{\Omega }{2u_{gb}}=\left(-\Delta G\mp \frac{\left(v_{gb}+S\right)}{2u_{gb}}\right)\Omega$$

Comparison of Equations (142) and (143) gives $M_{face}^{\left(1,2\right)}=M_{edge}^{\left(1,2\right)}$. Hence, calculation of the chemical potential distribution is completely self-consistent, as schematically represented in Figure 11.

## Appendix D. Retarding Effect of the Peripheral Toroid on the Moving Grain Boundary

In this Appendix the retarding effect of the peripheral toroid on moving grain boundary is calculated. Geometry factors of the moving toroid should be chosen in such a way that the drag force exerted on grain boundary by the toroid concides with that exerted by the full set of the peripheral bubbles. Superposition of Equations (91), (92), (94), (95), (97), (98), (112), (114), (121) and (123) with the ideal gas state equation forms the system of 10 equations:

(144) $$\left\{ \begin{array}{l} F_{tor}=\left(\Delta G+2\varepsilon_{tor}\right)S_{tor},\\ \varepsilon_{tor}=\frac{\gamma_{S}}{b_{2}}-\frac{\sin\theta_{2}\gamma_{S}}{c+b_{2}(\cos\theta_{2}\cot\phi -\sin\theta_{2})}-p_{tor},\\ v_{gb}=u_{gb}\left(\Delta G-F_{f}n_{f}-F_{e}n_{e}-F_{c}n_{c}\right),\\ F_{tor}=\left(F_{e}n_{e}+F_{c}n_{c}\right)S_{face},\\ v_{gb}=F_{f}\frac{D_{gb}w\chi \Omega }{kT\pi \rho_{b}^{3}},\\ v_{gb}=\frac{D_{gb}\Omega }{kT}\left(\Delta G+2\varepsilon_{tor}\right){\left[\frac{S_{e}N_{e}}{\pi e\eta_{e}}+2w\left(1+\frac{\rho_{b}^{2}}{R_{c}^{2}}\right)\right]}^{-1},\\ \frac{b_{1}\sin\left(\theta_{1}+\phi -\pi /2\right)}{\sin(\pi -\phi )}=\frac{b_{2}\sin\left(\theta_{2}+\phi -\pi /2\right)}{\sin(\pi -\phi )},\\ V\left(b_{1},c_{1},\phi ,\theta \right)+V\left(b_{2},c_{2},\phi ,\theta \right)=2N_{e}V_{e}+2N_{c}V_{c},\\ \cos\theta_{1}+\cos\theta_{2}=\gamma_{gb}/\gamma_{S},\\ p_{tor}V_{tor}=p_{tor}^{\left(0\right)}V_{tor}^{\left(0\right)}, \end{array}\right.$$

with 11 unknowns: *v_gb_*, *F_f_*, *F_e_*, *F_c_*, *F_tor_*, *b*_1_, *b*_2_, *θ*_1_, *θ*_2_, *p_tor_*, *ε_tor_*. However, *F_e_* and *F_c_* enter in the system only in combination $F_{e}n_{e}+F_{c}n_{c}=F_{tor}/S_{face}$. Consequently, solution of Equation (144) has the form:

(145) $$v_{gb}=u_{gb}\Delta G{\left[1+u_{gb}{\left(\frac{D_{gb}\Omega }{kT\pi \rho_{b}^{3}}\right)}^{-1}n_{f}+u_{gb}\left(\frac{S_{e}N_{e}}{\pi e\eta_{e}}+2w\left(1+\frac{\rho_{b}^{2}}{c^{2}}\right)\right)\cdot {\left(\frac{D_{gb}\Omega }{kT}\right)}^{-1}\frac{S_{tor}}{S_{face}}\right]}^{-1}$$
